# Synthetic single domain antibodies for the conformational trapping of membrane proteins

**DOI:** 10.7554/eLife.34317

**Published:** 2018-05-24

**Authors:** Iwan Zimmermann, Pascal Egloff, Cedric AJ Hutter, Fabian M Arnold, Peter Stohler, Nicolas Bocquet, Melanie N Hug, Sylwia Huber, Martin Siegrist, Lisa Hetemann, Jennifer Gera, Samira Gmür, Peter Spies, Daniel Gygax, Eric R Geertsma, Roger JP Dawson, Markus A Seeger

**Affiliations:** 1 Institute of Medical MicrobiologyUniversity of ZurichZurichSwitzerland; 2 Roche Pharma Research and Early DevelopmentTherapeutic Modalities, Roche Innovation Center Basel, F. Hoffmann-La Roche LtdBaselSwitzerland; 3 University of Applied Sciences and Arts Northwestern SwitzerlandMuttenzSwitzerland; 4 Institute of BiochemistryGoethe University FrankfurtFrankfurt am MainGermany; NINDSUnited States

**Keywords:** membrane protein, nanobody, ribosome display, phage display, in vitro selection, conformational trapping, *E. coli*

## Abstract

Mechanistic and structural studies of membrane proteins require their stabilization in specific conformations. Single domain antibodies are potent reagents for this purpose, but their generation relies on immunizations, which impedes selections in the presence of ligands typically needed to populate defined conformational states. To overcome this key limitation, we developed an in vitro selection platform based on synthetic single domain antibodies named sybodies. To target the limited hydrophilic surfaces of membrane proteins, we designed three sybody libraries that exhibit different shapes and moderate hydrophobicity of the randomized surface. A robust binder selection cascade combining ribosome and phage display enabled the generation of conformation-selective, high affinity sybodies against an ABC transporter and two previously intractable human SLC transporters, GlyT1 and ENT1. The platform does not require access to animal facilities and builds exclusively on commercially available reagents, thus enabling every lab to rapidly generate binders against challenging membrane proteins.

## Introduction

Conformation-specific binders raised against membrane proteins have the ability to manipulate cells directly at the cell surface and are exquisite tools for basic science and drug discovery (Ahuja et al., 2015; Blanpain et al., 2002; Lee et al., 2013). However, binder selection against this difficult class of target proteins is very challenging (Dominik et al., 2016; Doshi et al., 2014; Pardon et al., 2014), because membrane proteins tend to be flexible, exhibit only small hydrophilic surfaces, require detergents or lipids to remain folded and are typically obtained only in small amounts. A particularly successful method to generate binders against membrane proteins relies on the immunization of camelids for the pre-enrichment of B-cells that encode target-specific heavy-chain-only antibodies, whose variable domains are called VHH or nanobodies (Pardon et al., 2014). The success of nanobodies is rooted in the simplicity and robustness of the VHH scaffold and in its characteristic variability at the complementarity determining region 3 (CDR3), which is frequently found to penetrate deeply into cavities of membrane protein targets (Kruse et al., 2013; Rasmussen et al., 2011a). CDR3 loops of variable length and orientation create diverse binder shapes, thus permitting an optimal surface-complementarity to antigens.

Despite of the outstanding track record of camelid nanobodies, there are three major restrictions linked to immunizations. First, the target space is limited to comparatively stable proteins, because delicate targets (e.g. many human membrane transporters) readily unfold upon injection due to the applied adjuvants and the camelid’s high body temperature. Second, it is very difficult to favor target conformations with non-covalent ligands because they dissociate from the protein shortly after injection, unless their affinities are extremely high, as was the case for the β2 adrenergic receptor agonist BI-167107 having a dissociation constant of as low as 84 pM (Rasmussen et al., 2011a). Third, immunizations require access to animal facilities, which makes it difficult and expensive for many researchers to implement binder generation on a routine basis in their own lab.

Pure in vitro binder selection methods in theory can overcome these drawbacks of immunization, because they allow for the full control over the binder selection process. However, in praxis there are only a few published examples from specialized labs reporting on successful in vitro selections against integral membrane proteins (Dominik et al., 2016; Kodan et al., 2014; Seeger et al., 2012; Stockbridge et al., 2014; Uysal et al., 2009). This stands in contrast to many prominent examples of membrane protein binders that resulted from immunizations (Ehrnstorfer et al., 2014; Huang et al., 2015; Krishnamurthy and Gouaux, 2012; Liang et al., 2017; Rasmussen et al., 2011b). Potential limitations of synthetic binders are (i) small library sizes (namely 10^9^–10^10^ for phage or yeast display), which can result in weak binding affinities (ii) sub-optimal framework design, which can give rise to aggregated or poorly expressing library members and (iii) selection bias as a consequence of binder display and target immobilization, which can lead to poorly enriched binder pools (Bradbury et al., 2011). These shortcomings in particular impede in vitro binder selections against membrane proteins. Consequently, extensive binder screening and purification efforts are often required after selection to identify suitable binders, as was for example the case for DARPin selections against the ABC transporters MsbA and LmrCD carried out in our lab (Mittal et al., 2012; Seeger et al., 2012).

In this work, we introduce a selection platform, tailored to tackle membrane protein targets, which overcomes current limitations of immunizations and in vitro selections. At the core of our technology are highly stable synthetic scaffolds called sybodies, which are designed to mimic the natural shape diversity of camelid nanobodies, thus allowing for an optimal surface complementarity to the limited hydrophilic epitopes on membrane proteins. The application of ribosome display for synthetic nanobody libraries allows processing of very large diversities (Binz et al., 2004; Hanes and Plückthun, 1997), thus compensating for the incremental antibody maturation taking place in vivo. Our approach permits the selection and preparative production of sybodies within three weeks and requires only standard laboratory materials. In order to validate our platform, we generated conformation-selective sybodies against two previously intractable, disease-relevant human SLC transporters binding to their inhibitor-locked states and trapped an ABC transporter in its transient ATP-bound conformation.

## Results

### The three binding modes of sybodies

**Figure 1.**
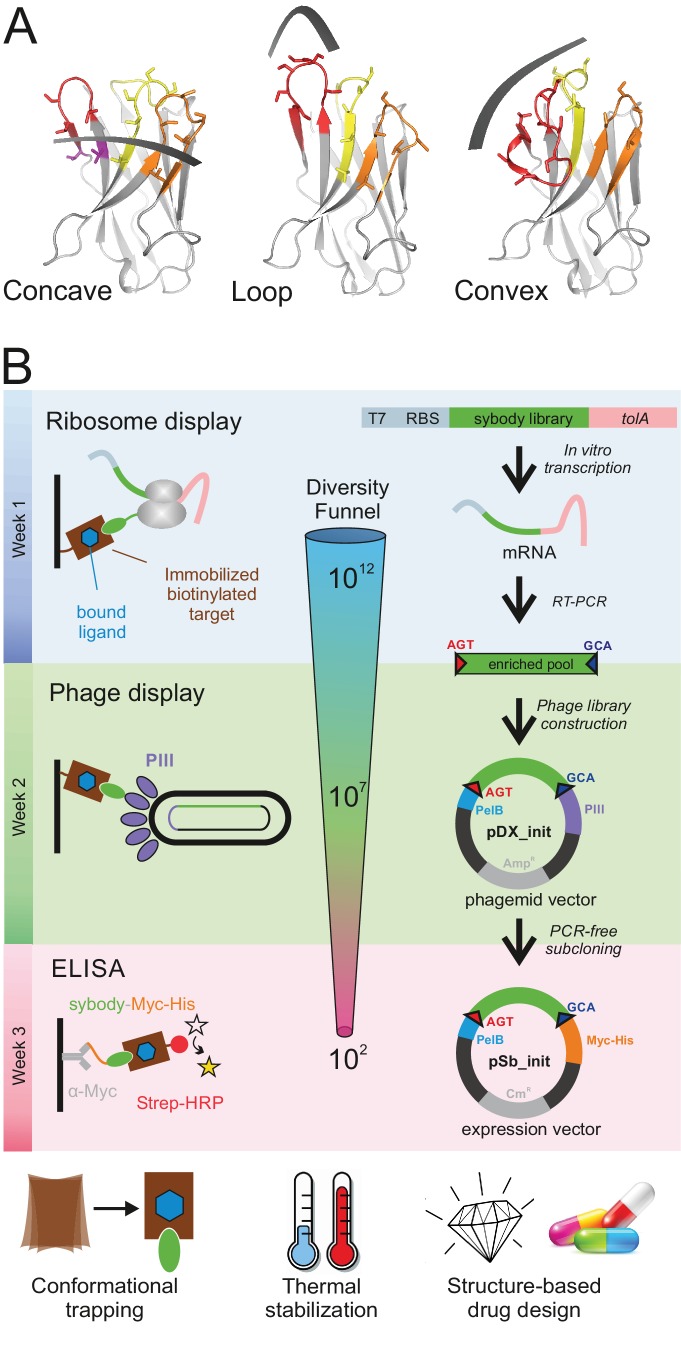
Selection of sybodies against membrane proteins within three weeks. (**A**) Three synthetic libraries exhibiting highly variable randomized surfaces (concave, loop and convex) each harboring a diversity of 9 × 10^12^ were designed based on thermostabilized nanobody frameworks. CDR1, CDR2 and CDR3 are colored in yellow, orange and red, respectively. (**B**) The in vitro selection platform is built as a selection cascade, starting with 10^12^ sybodies displayed on ribosomes for pre-enrichment, followed by a focused phage display library of 10^7^ clones and binder identification by ELISA (typically 96 clones). The platform builds on fragment exchange (FX) cloning using Type IIS restriction sites encoded on the phage display (pDX_init) and expression vector (pSb_init) backbones, which generate AGT and GCA sticky ends for PCR-free subcloning. Key elements for reliable selections against membrane proteins are the shape variability of the sybody libraries, exceptionally high experimental diversities using ribosome display and the change of display system during the selection process.

We analyzed a large number of deposited camelid nanobody structures and found that different lengths and arrangements of the CDR3 results in three groups of interaction surfaces, which can be described as concave, loop and convex. In order to mimic the surface complementarity repertoire of the camelid immune system and its capacity to efficiently target membrane proteins, we designed three sybody libraries based on prototypical camelid nanobody structures representing these three binding modes. The first template nanobody is a GFP-binder with a short CDR3 (six amino acids (aa) according to [Sircar et al., 2011]) that binds via a concave surface (PDB: 3K1K) (Kirchhofer et al., 2010). The second template is a β_2_-adrenergic receptor binder that inserts a medium length CDR3 (12 aa) as an extended loop into a receptor cavity (PDB: 3P0G) (Rasmussen et al., 2011a). The third template is a lysozyme binder displaying a long CDR3 (16 aa), which is tethered via an extended hydrophobic core, and binds via a convex surface (PDB: 1ZVH) (De Genst et al., 2006) (Figure 1, Figure 1—figure supplement 1, Table 1). The resulting sybody libraries were therefore dubbed ‘concave’, ‘loop’ and ‘convex’, accordingly.

**Figure 1—figure supplement 1.**
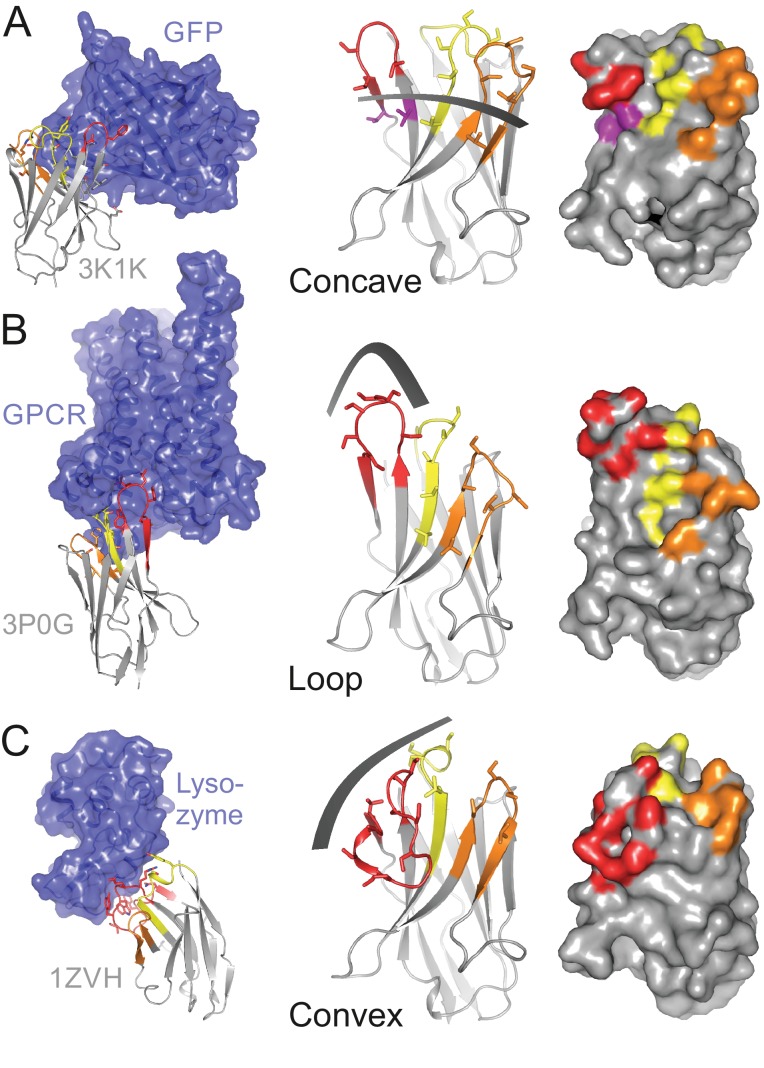
Variable sybody scaffolds based on three camelid nanobodies. CDR1, CDR2 and CDR3 are colored in yellow, orange and red, respectively. In the left panel, crystal structures of camlid nanobodies in complex with GFP (PDB: 3K1K) (**A**), a GPCR (PDB: 3P0G) (**B**) and Lysozyme (PDB: 1ZVH) (**C**) are shown, which served as starting point to delineate scaffolds for randomization. Nanobody residues contacting the target proteins are depicted as sticks. The target proteins are colored in blue. In the middle panel, homology models of three framework nanobodies are shown as cartoons and randomized residues (defined as serines and threonines in these examples) are highlighted as sticks. The three sybody libraries exhibit a concave (**A**), loop (**B**) or convex (**C**) binding surface, respectively. The right panel shows the randomized surface of the three libraries with the side chains of the randomized positions highlighted in color. Note that the concave library contains randomized residues outside of the CDR regions, which are colored in purple.

**Figure 1—figure supplement 2.**
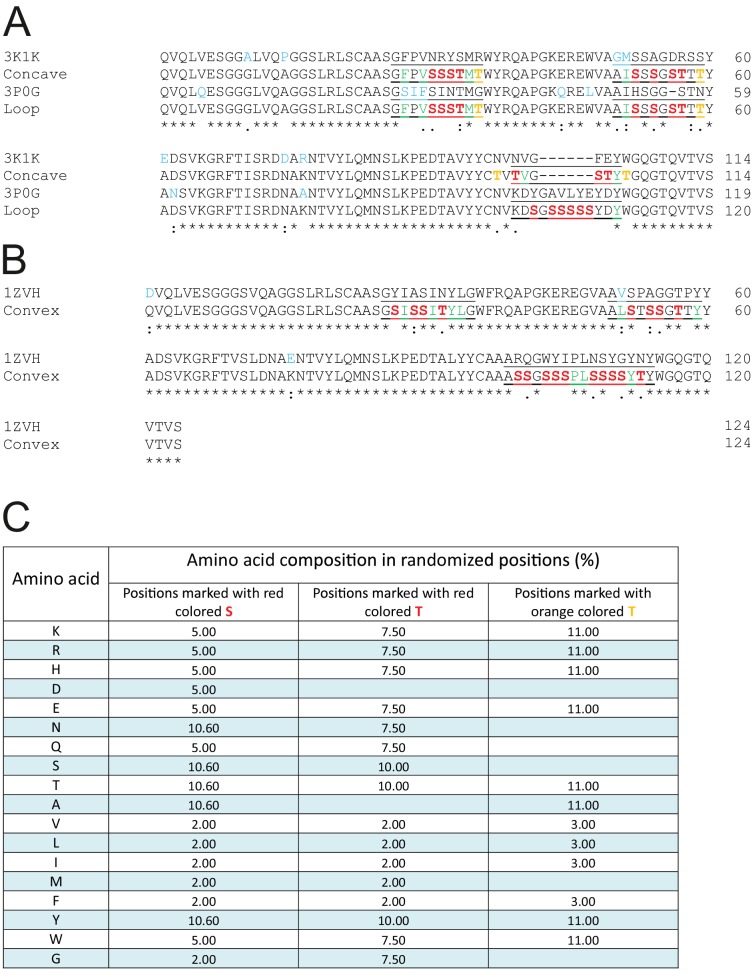
Framework sequences and randomized positions. (**A** and **B**) Sequences of the framework sybodies are aligned with the sequences of their natural precursors. The frameworks of the concave and the loop library are identical (**A**) while the convex library has its own scaffold (**B**). Residues of the natural precursor nanobodies differing from the framework sequence are marked in blue. The three CDR regions are underlined. Invariant CDR residues contributing to the hydrophobic core of the respective scaffold are marked in green. Note that the differently shaped libraries exhibit alternative sets of invariant CDR residues that precisely match the corresponding scaffolds. This harmonization is a critical and unprecedented feature of our synthetic nanobdy libraries, as it allows for the first time to include variable CDR lengths without the risk of scaffold destabilization. Randomized residues are highlighted as red S (for which randomization mixture one was used), as red T (mix 2) and orange T (mix 3). (**C**) Amino acid composition of randomized positions obtained by three different trinucleotide randomization mixtures. The rationale behind the three randomization mixtures is provided in the main text.

**Figure 1—figure supplement 3.**
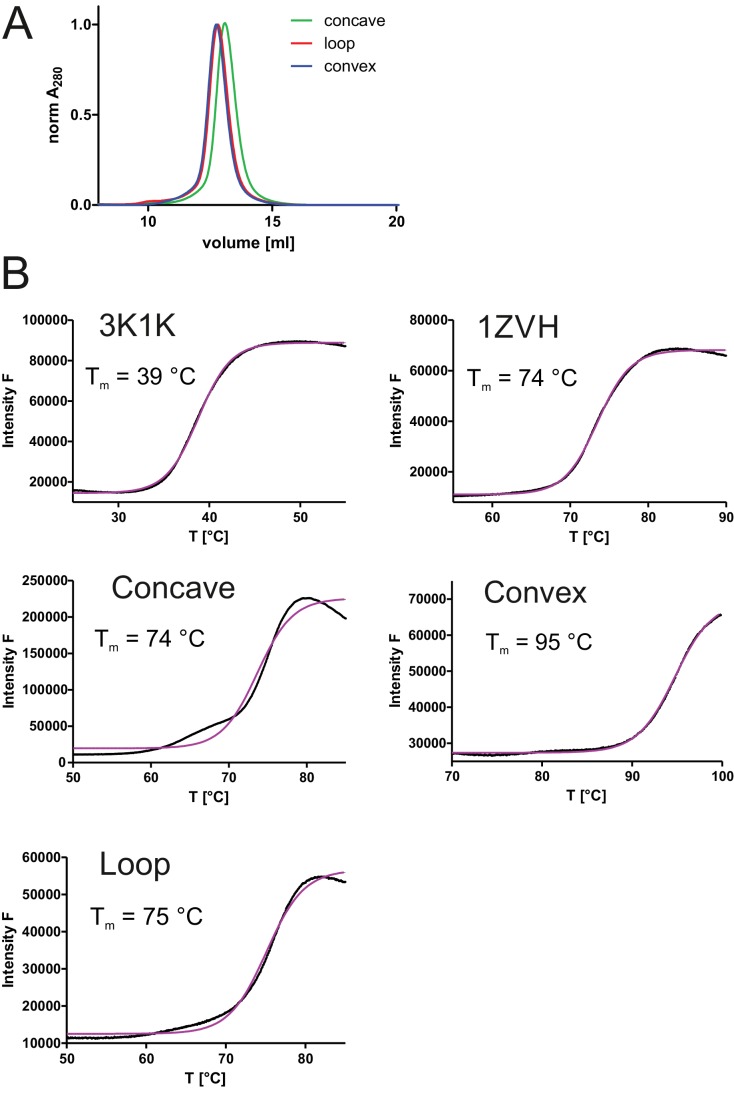
Biophysical characterization of sybodies. Three framework sybodies representing the concave, the loop and the convex library and containing serines and threonines in the randomized positions were generated by gene synthesis (sequences provided in Figure 1—figure supplement 2). (**A**) SEC analysis of periplasmatically expressed concave, loop and convex framework sybodies using a Superdex 75 300/10 GL column. (**B**) Determination of melting temperature (*T*_m_) of framework sybodies and their natural precursors 3K1K and 1ZVH using dye SYPRO Orange (ThermoFluor). Representative data of two technical replicates are shown.

**Figure 1—figure supplement 4.**
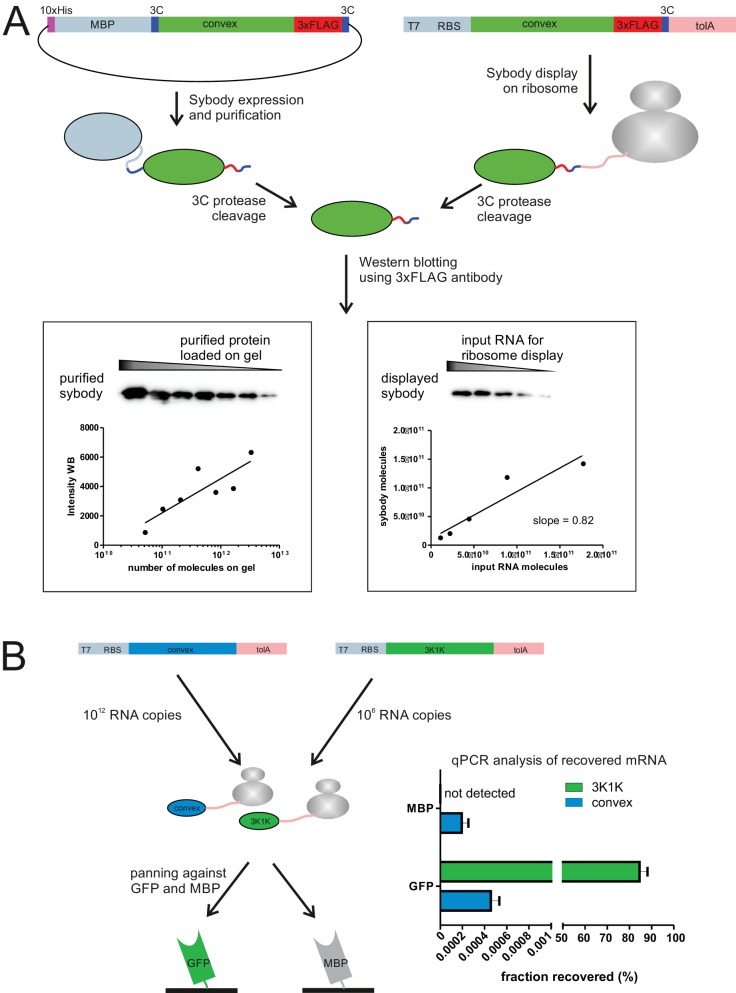
Ribosome display of single domain antibodies. (**A**) The non-randomized convex sybody was either purified containing a C-terminal 3x-FLAG tag or displayed on ribosomes containing the same tag using the commercial kit PURE*frexSS* (GeneFrontier). 3C protease cleavage was used to liberate the displayed sybody from the ribosomal complex. Western blotting analysis using anti-3x-FLAG antibody and purified sybody as standard revealed a display efficiency of 82% of input mRNA for ribosome display. (**B**) 10^6^ mRNA molecules encoding the GFP-specific 3K1K nanobody were displayed on ribosomes using PURE*frexSS* together with 10^12^ mRNA molecules encoding the non-randomized convex sybody. The ribosomal complexes were pulled down using either biotinylated GFP or MBP immobilized on magnetic beads. The mRNA of isolated ribosomal complexes was isolated, reverse transcribed and the resulting cDNA was analyzed by qPCR performing technical triplicates. This analysis revealed that 84.6 ± 3.5% (error corresponds to standard deviation) of the input 3K1K mRNA was retrieved on GFP-coated beads, while virtually no background binding of the non-randomized convex sybody nor 3K1K binding to MBP was observed.

**Figure 1—figure supplement 5.**
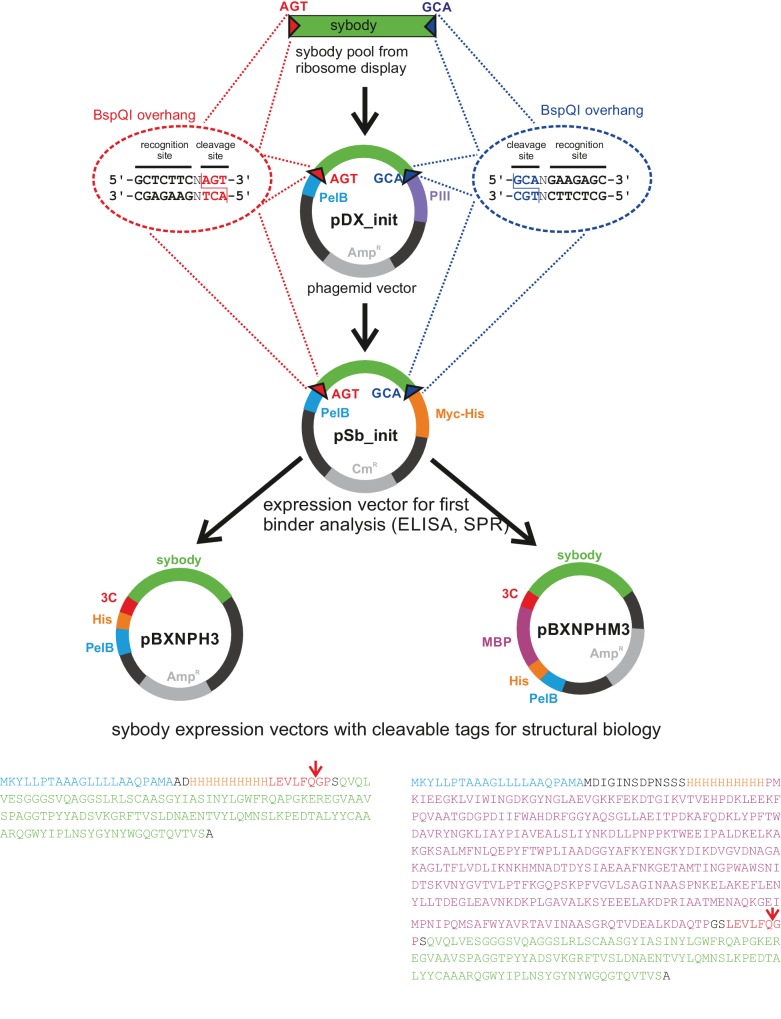
FX cloning vector series for phage display and purification of sybodies and nanobodies. Sybody pools from ribosome display (or nanobodies from immunized camelids) are amplified with primers containing restriction sites of Type IIS enzyme BspQI (isoschizomer of SapI) to generate AGT and GCA overhangs. BspQI restriction sites generating the same overhangs were introduced into the backbones of vector pDX_init for phage display and pSb_init for periplasmatic expression and attachment of Myc- and His-tag. Note that in pDX_init and pSb_init the BspQI restriction sites are part of the sybody open reading frame. Finally, sybodies/nanobodies are sub-cloned from pSb_init to the destiny vectors pBXNPH3 or pBXNPHM3 for periplasmic expression. Tag-less sybodies/nanobodies for structural biology purposes can be obtained by 3C protease cleavage. Importantly, the vector series permits for PCR-free subcloning once the sybodies have been inserted into phage display vector pDX_init. The vectors were made available through Addgene (for Addgene IDs, see Table 3).

**Figure 1—figure supplement 6.**
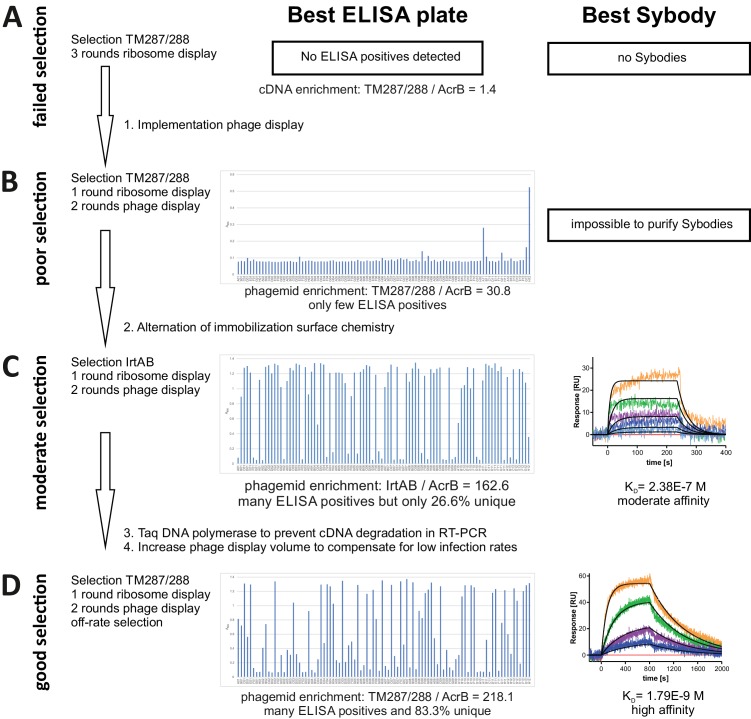
Improvement of the sybody selection procedure. (**A**) Three rounds of ribosome display using the same type of magnetic beads for target immobilization (Dynabeads Myone Streptavidin T1) failed to generate sybodies against ABC transporter TM287/288. Pool enrichment against TM287/288 compared to negative control AcrB was poor. No positive ELISA hits were identified. (**B**) Sybody selections against TM287/288 were performed applying one round of ribosome display followed by two rounds of phage display using Dynabeads Myone Streptavidin T1 for target immobilization. The pool was enriched approximately 30 fold and a few positive ELISA hits were found. Purification of identified sybodies failed. (**C**) Sybody selections against ABC transporter IrtAB, a homologue of TM287/288 sharing a sequence identity of 27%, was performed as in (**B**), but using different immobilization chemistries (Dynabeads Myone Streptavidin T1 for ribosome display, Maxisorp microtiter plates for the first phage display round and Dynabeads Myone Streptavidin C1 for the second phage display round) to suppress accumulation of background binders. Strong enrichment was observed and a high number of positive ELISA hits were identified. Only 27% of positive ELISA hits were unique sybodies with moderate affinities. (**D**) Final optimized sybody selection protocol as described in the materials and methods section. Diversity bottlenecks were removed by using Taq DNA polymerase for cDNA amplification and increasing the working volume of the first phage display round. An off-rate selection step was introduced in the second phage display round. Enrichment and number of ELISA hits was similar to the selection shown in (**C**). The number of unique ELISA hits increased to 83% and high affinity binders were obtained. The binders obtained in (**D**) against TM287/288 are described in detail in main Figures 3 and 4.

**Table 1.** Features of the three sybody libraries.

Library	Template PDB entry/target	Binding interface in template	Length of CDR3	Number of randomized residues in library	Theoretical diversity of library
concave	3K1K/GFP	672 Å^2^	6 aa	15	8.3 × 10^17^
loop	3P0G/GPCR	901 Å^2^	12 aa	16	4.3 × 10^19^
convex	1ZVH/Lysozyme	533 Å^2^	16 aa	18	2.8 × 10^22^

### Establishment of the sybody framework and randomization strategy

Apart from the CDR regions, 3K1K and 3P0G share high sequence identities and therefore the concave and the loop library share the same framework (Figure 1—figure supplement 2). In contrast, 1ZVH contains an extended hydrophobic core and the convex library was therefore built on a different scaffold (Figure 1—figure supplement 2). A single, conserved disulfide bond at the center of the immunoglobulin domain is common to all three scaffolds. Three non-randomized scaffold sybodies representing the concave, the loop and the convex library were generated by gene synthesis. They contain serines and threonines at positions to be randomized in the libraries (Table 2, Figure 1—figure supplement 2). The corresponding purified proteins eluted as a single species from a size exclusion chromatography column (Figure 1—figure supplement 3A). They exhibited high melting temperatures of 74, 75 and 95°C for the concave, loop and convex scaffold, respectively, which corresponds to a stability increase of 21 to 35°C compared to their natural precursors (Figure 1—figure supplement 3B). Thermal stability of the convex sybody is particularly high. We attributed this increased stability to the extended hydrophobic core to tether CDR3 and the V51L substitution introduced into the framework prior to the CDR2 region.

**Table 2.** DNA sequences of non-randomized sybodies and flanking regions for ribosome display

Framework sequence concave	CAGGTTCAGCTGGTTGAGAGCGGTGGTGGCCTGGTCCAAGCTGGCGGTTCGCTGCGTCTGAGCTGCGCCGCAAGCGGTTT CCCGGTGAGCAGCAGCACGATGACCTGGTATCGTCAGGCACCGGGCAAAGAACGTGAGTGGGTCGCGGCGATTTCCAGCT CTGGTAGCACCACGACCTACGCAGATTCTGTTAAGGGCCGCTTTACCATCAGCCGCGACAACGCGAAGAATACGGTCTAT TTGCAGATGAATAGCCTGAAACCGGAAGATACCGCGGTTTACTACTGTACCGTGACCGTGGGTAGCACGTACACGGGCCA AGGTACCCAAGTGACTGTGAGC

Framework sequence loop	CAGGTTCAGCTGGTTGAGAGCGGTGGTGGCCTGGTCCAAGCTGGCGGTTCGCTGCGTCTGAGCTGCGCCGCAAGCGGTTT CCCGGTGAGCAGCAGCACGATGACCTGGTATCGTCAGGCACCGGGCAAAGAACGTGAGTGGGTCGCGGCGATTTCCAGCT CTGGTAGCACCACGACCTACGCAGATTCTGTTAAGGGCCGCTTTACCATCAGCCGCGACAACGCGAAGAATACGGTCTAT TTGCAGATGAATAGCCTGAAACCGGAAGATACCGCGGTTTACTACTGTAACGTGAAAGACAGCGGTAGCTCCAGCAGCTC CTACGACTATTGGGGCCAAGGTACCCAAGTGACTGTGAGC
Framework sequence convex	CAAGTCCAGCTGGTGGAATCGGGTGGTGGTAGCGTCCAGGCGGGTGGTAGCCTGCGTCTGAGCTGTGCGGCTAGCGGCTC TATTTCCAGCATCACGTACCTGGGCTGGTTTCGCCAGGCACCGGGCAAAGAGCGTGAGGGCGTCGCAGCGCTGAGCACCA GCTCCGGTACCACCTACTACGCGGACAGCGTTAAGGGTCGTTTCACGGTGAGCCTGGACAACGCCAAGAATACCGTGTAT CTGCAAATGAACAGCTTGAAACCGGAAGATACTGCTTTGTATTACTGCGCGGCAGCCAGCAGCGGCTCCAGCAGCCCGCT GTCTAGCAGCAGCTATACGTACTGGGGTCAGGGCACCCAAGTTACCGTTTCT
5’ flank ribosome display	TAATACGACTCACTATAGGGAGACCACAACGGTTTCCCTCTAGAAATAATTTTGTTTAACTTTAAGAAGGAGATATATCC ATGGGTAGT
3’ flank ribosome display	GCAAAGCTTTATATGGCCTCGGGGGCCGAATTCGGATCTGGTGGCCAGAAGCAAGCTGAAGAGGCGGCAGCGAAAGCGGC GGCAGATGCTAAAGCGAAGGCCGAAGCAGATGCTAAAGCTGCGGAAGAAGCAGCGAAGAAAGCGGCTGCAGACGCAAAGA AAAAAGCAGAAGCAGAAGCCGCCAAAGCCGCAGCCGAAGCGCAGAAAAAAGCCGAGGCAGCCGCTGCGGCACTGAAGAAG AAAGCGGAAGCGGCAGAAGCAGCTGCAGCTGAAGCAAGAAAGAAAGCGGCAACTGAAGCTGCTGAAAAAGCCAAAGCAGA AGCTGAGAAGAAAGCGGCTGCTGAAAAGGCTGCAGCTGATAAGAAAGCGGCAGCAGAGAAAGCTGCAGCCGACAAAAAAG CAGCAGAAAAAGCGGCTGCTGAAAAGGCAGCAGCTGATAAGAAAGCAGCGGCAGAAAAAGCCGCCGCAGACAAAAAAGCG GCAGCGGCAAAAGCTGCAGCTGAAAAAGCCGCTGCAGCAAAAGCGGCCGCAGAGGCAGATGATATTTTCGGTGAGCTAAG CTCTGGTAAGAATGCACCGAAAACGGGGGGAGGGGCGAAAGGGAACAATGCTTCGCCTGCCGGGAGTGGTAATACTAAAA ACAATGGCGCATCAGGGGCCGATATCAATAACTATGCCGGGCAGATTAAATCTGCTATCGAAAGTAAGTTCTATGACGCA TCGTCCTATGCAGGCAAAACCTGTACGCTGCGCATAAAACTGGCACCCGATGGTATGTTACTGGATATCAAACCTGAAGG TGGCGATCCCGCACTTTGTCAGGCTGCGTTGGCAGCAGCTAAACTTGCGAAGATCCCGAAACCACCAAGCCAGGCAGTAT ATGAAGTGTTCAAAAACGCGCCATTGGACTTCAAACCGTAG

Based on these scaffolds, the three sybody libraries were constructed by randomizing all three CDRs using defined mixtures of trinucleotides, thereby obtaining an optimal balance between charged, polar, aromatic and apolar amino acids to achieve an overall moderate hydrophobicity of the randomized surface (Figure 1—figure supplement 1). Decreased surface hydrophobicity was previously demonstrated in a DARPin library to be of paramount importance to counteract the enrichment of sticky binders when selecting against membrane proteins (Seeger et al., 2013). Three trinucleotide mixes were used for randomized residues placed (i) in loops, (ii) at the transitions from loops to β-sheets and (iii) in the middle of β-sheets (Figure 1—figure supplement 2C). Cysteines and prolines were generally excluded in any of the three mixes. Mix one is enriched by the residues A, S, T, N, Y (10.6%, each), contains D, E, Q, R, K, H, W at 5% frequency each and harbors only few of the apolar amino acids F, M, V, I, L, G (2%, each). Mix two lacks amino acids D and A, because these two residues are underrepresented at the end of β-sheets (Bhattacharjee and Biswas, 2010). Mix three is devoid of D, N, Q, G, S and M, because these amino acids are found less frequently in the middle of β-sheets (Bhattacharjee and Biswas, 2010). The theoretical diversity of the libraries amounts to 8.3 × 10^17^, 4.3 × 10^19^, and 2.8 × 10^22^ for the concave, loop and convex library, respectively. Three DNA fragments each containing one CDR of each of the three libraries were generated by assembly PCR. The resulting fragments were ligated in two subsequent steps using Type IIS restriction enzymes analogous to the assembly of designed ankyrin repeat proteins (Seeger et al., 2013). Finally, the three sybody libraries were flanked with the required sequence elements for in vitro transcription and ribosome display (Table 2). We determined an experimental library diversity of 9 × 10^12^ for each of the three libraries.

### Ribosome display of sybodies and nanobodies

Provided that libraries of high quality are used, binder affinities obtained by in vitro selections largely depend on the displayed library size in the initial selection round, because other than in the animal, no affinity maturation is performed. In contrast to the widely used phage and yeast display systems, which have maximal library sizes of 10^9^–10^10^ members, ribosome display offers the advantage of displaying 10^12^ different library members with minor experimental effort. In ribosome display, a stable ternary complex between an encoding mRNA, the ribosome and the folded nascent polypeptide chain is formed. However, ribosome display is not widely used, because it used to require the preparation of home-made in vitro translation reagents and is associated with variable levels of unfavorable RNase acitivity (Zahnd et al., 2007). To overcome this technical hurdle which prevented non-expert labs from using this efficient display method, we implemented the commercial in vitro translation kit PURE*frexSS* (GeneFrontier) for ribosome display. The kit is devoid of reducing agents and contains oxidized glutathione (GSSG) and the disulfide bond isomerase DsbC and is thus suited to support the folding of disulfide-containing proteins such as nanobodies and sybodies. We experimentally tested display efficiency in two independent assays. In a first assay, we fused a 3xFLAG tag followed by 3C protease cleavage site to the C-terminus of the non-randomized loop sybody in a construct containing the flanking regions for transcription and ribosome display (Figure 1—figure supplement 4A). The mRNA of this construct was displayed on ribosomes using the PURE*frexSS* kit, sybody-3xFLAG was cleaved from the nascent polypeptide chain by 3C protease and the entire protein mixtures was analyzed by Western blotting using an anti-FLAG antibody. A purified non-randomized convex sybody containing exactly the same 3xFLAG sequence and 3C protease cleavage site at the C-terminus served as standard for protein quantification by Western blotting. The analysis revealed that more than 70% of the input mRNA was translated. In a second assay, we examined whether ribosome display produces correctly folded binders. To this end, we displayed 10^6^ mRNA encoding the 3K1K nanobody spiked into 10^12^ mRNA encoding the non-randomized convex sybody using PURE*frexSS* kit and assessed binding to immobilized GFP (target of 3K1K) and MBP (negative control) (Figure 1—figure supplement 4B). Quantitative PCR on reverse transcribed cDNA was used to determine the amount of mRNA which could be retrieved after binding and washing in comparison to the input mRNA added to the display reaction. For 3K1K panned against GFP, mRNA recovery was 84.6 ± 3.5%, while mRNA retrieved from 3K1K panning against MBP was not detectable. Background binding of the non-randomized convex sybody towards GFP and MBP was minimal (recovery fractions below 0.001%). These two independent experiments clearly demonstrated that ribosome display of single domain antibodies works efficiently using the commercial PURE*frexSS* kit.

### Sybody selections against maltose binding protein

**Figure 2.**
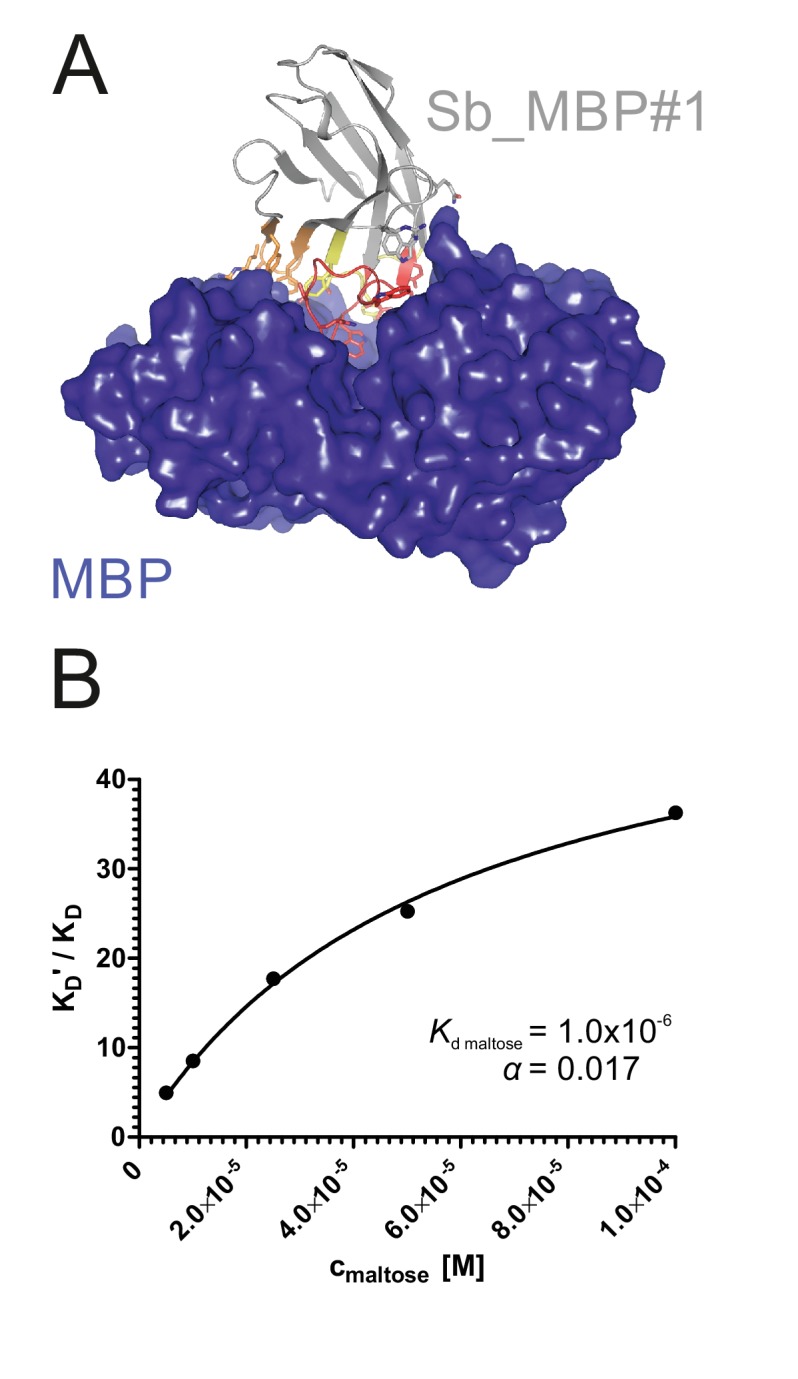
Structural and biochemical characterization of convex sybody Sb_MBP#1. (**A**) Crystal structure of the Sb_MBP#1/MBP complex. MBP is shown as blue surface, the convex sybody Sb_MBP#1 is shown as grey cartoon with CDRs 1–3 colored in yellow, orange and red, respectively. Sybody residues mediating contacts to MBP are shown as sticks. (**B**) Maltose and sybody Sb_MBP#1 compete for binding to MBP. In the depicted Schild analysis, the sybody affinity ratios determined in the presence (*K*_D_’) and absence (*K*_D_) of maltose is plotted against the maltose concentration. The binding affinity for maltose K_D,maltose_ was determined as 1.0 µM. The allosteric constant α amounts to 0.017, that is the ratio *K*_D_’/*K*_D_ saturates at a value of 58.

To validate the sybody libraries, first selections encompassing three consecutive rounds of ribosome display were carried out against the soluble maltose binding protein (MBP), which can be considered as easy target (Figure 2—figure supplement 1). Sybody pools were found to be strongly enriched after the third selection round as monitored by qPCR. Using FX cloning (Geertsma and Dutzler, 2011), single sybodies were introduced into expression vector pSb_init, which directs the protein into the periplasm by virtue of a PelB leader sequence and adds a Myc- and a His-tag to the C-terminus of the sybody for detection by ELISA (Table 3, Figure 1—figure supplement 5). Of note, pSb_init contains BspQI restriction sites, which permits to release sybodies for sub-cloning into expression plasmids pBXNPH3 and pBXNPHM3 for the production of tag-less binders for crystallization purposes. ELISA analysis of the selections of the concave, loop and convex library revealed about 20, 50 and 30% of all wells as strong and specific hits against MBP. Crystal structures of three convex MBP binders (Sb_MBP#1–3) in complex with MBP were solved at resolutions ranging from 1.4 to 1.9 Å (Figure 2, Table 4). The structures of the crystallized sybodies were highly similar to their natural precursor (e.g. RMSD of 1.02 Å comparing Sb_MBP#1 and 1ZVH), thereby validating our library design, which kept selected residues of the CDRs constant to assure folding of an extended hydrophobic core (Figure 2—figure supplement 2). Sb_MBP#1–3 have binding affinities ranging from 24 to 500 nM as determined by surface plasmon resonance (SPR). They bind into the cleft between the two lobes of MBP and thereby trap the target in its ligand-free conformation (Figure 2A, Figure 2—figure supplement 3) (Spurlino et al., 1991). In support of this notion, SPR measurements revealed decreasing sybody binding affinities at increasing maltose concentrations. A Schild plot analysis revealed 58-fold decreased sybody binding affinity at saturating maltose concentration and an affinity of 1.0 µM of MBP for maltose (Figure 2B, Figure 2—figure supplement 4), which is in close agreement with the literature (Telmer and Shilton, 2003). An analysis of the binding interface highlighted CDR3 residues W101, Q104, S105 and W110, which are identical among the three binders (Figure 2—figure supplement 3). Further contacts are mediated by variable randomized residues of CDR1, CDR2 and CDR3 as well as several invariant framework residues. Of note, the sybody selections against MBP generated a highly variable set of binders from all three libraries exhibiting affinities down to 0.5 nM (Figure 2—figure supplement 1). These sybodies are expected to bind to a variety of epitopes on MBP, but were not further analyzed by crystallization trials. In summary, the crystallized sybodies bind to MBP in an analogous fashion as camelid nanobodies, namely via interactions predominantly mediated by CDR3 residues (De Genst et al., 2006; Desmyter et al., 2001).

**Figure 2—figure supplement 1.**
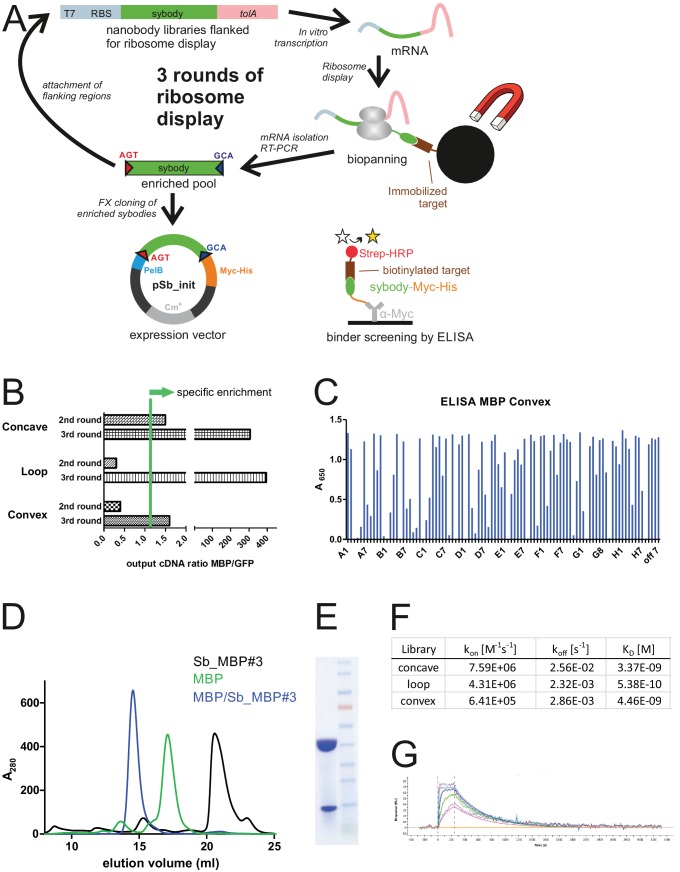
Sybody selections against MBP. (**A**) Sybodies were selected against MBP using three rounds of ribosome display and MBP immobilized on magnetic beads. Sybodies were expressed in pSb_init and analyzed by ELISA. (**B**) Binder enrichment was monitored using qPCR by comparing the cDNA output after panning against the target MBP versus the control protein GFP. (**C**) ELISA analysis of convex pool after selection round 3. MBP-specific DARPin off7 was used as positive control (Binz et al., 2004). (**D**) SEC analysis of sybody Sb_MBP#3 alone and in complex with MBP using a Superdex 200 300/10 GL column. (**E**) SDS-PAGE analysis of Sb_MBP#3/MBP complex after SEC. (**F**) K_D_, k_on_ and k_off_ values of the highest affinity sybodies obtained from the concave, loop and convex library, as measured by SPR. (**G**) SPR traces of the loop sybody exhibiting an affinity of 0.5 nM.

**Figure 2—figure supplement 2.**
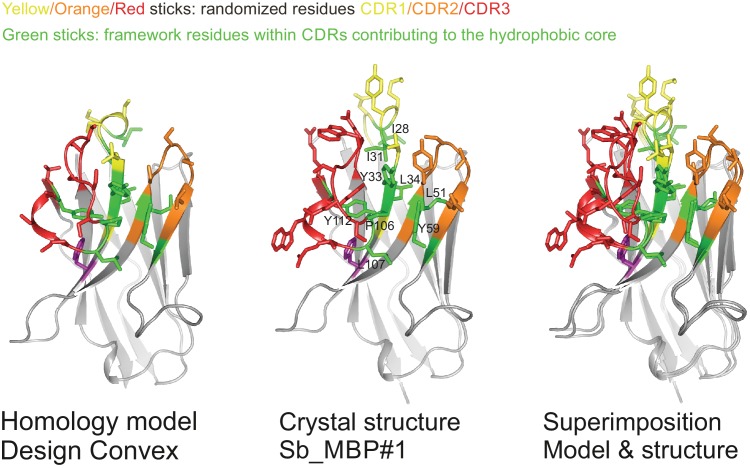
Validation of sybody library design. Comparison of homology model of non-randomized convex sybody based on the coordinates of 1ZVH with the structure of selected convex sybody Sb_MBP#1 (determined in complex with MBP). CDR residues contributing to the hydrophobic core are highlighted as green sticks, randomized residues as sticks colored in yellow, orange and red for CDR1, CDR2 and CDR3, respectively.

**Figure 2—figure supplement 3.**
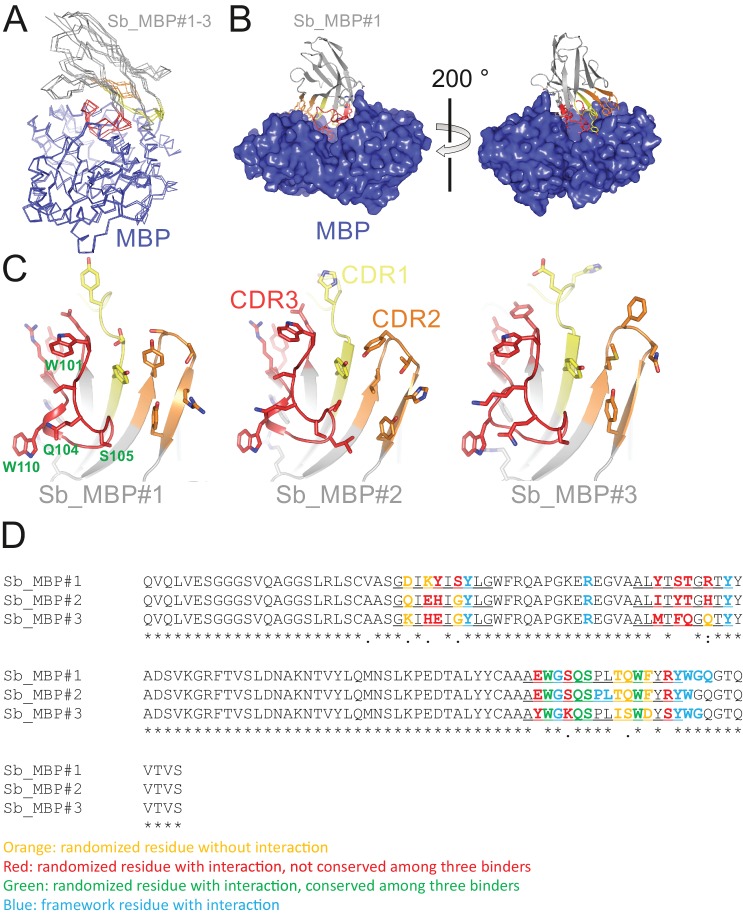
Detailed analysis of sybody-MBP complex structures. (**A**) Structures of MBP (blue) in complex with Sb_MBP#1–3 (grey with CDR1, CDR2 and CDR3 in yellow, orange and red, respectively). The coordinates of MBP were used to perform superimposition. (**B**) Interaction of Sb_MBP#1 with MBP, shown along the MBP cleft from both sides. Sybody residues contacting MBP (distance ≤4 Å) are shown as sticks. (**C**) Detailed view of interacting residues of sybodies Sb_MBP#1–3. In the left panel, four randomized residues of CDR3 which are invariant among the three binders are labeled. (**D**) Sequence alignment of Sb_MBP#1–3. The CDR regions are underlined.

**Figure 2—figure supplement 4.**
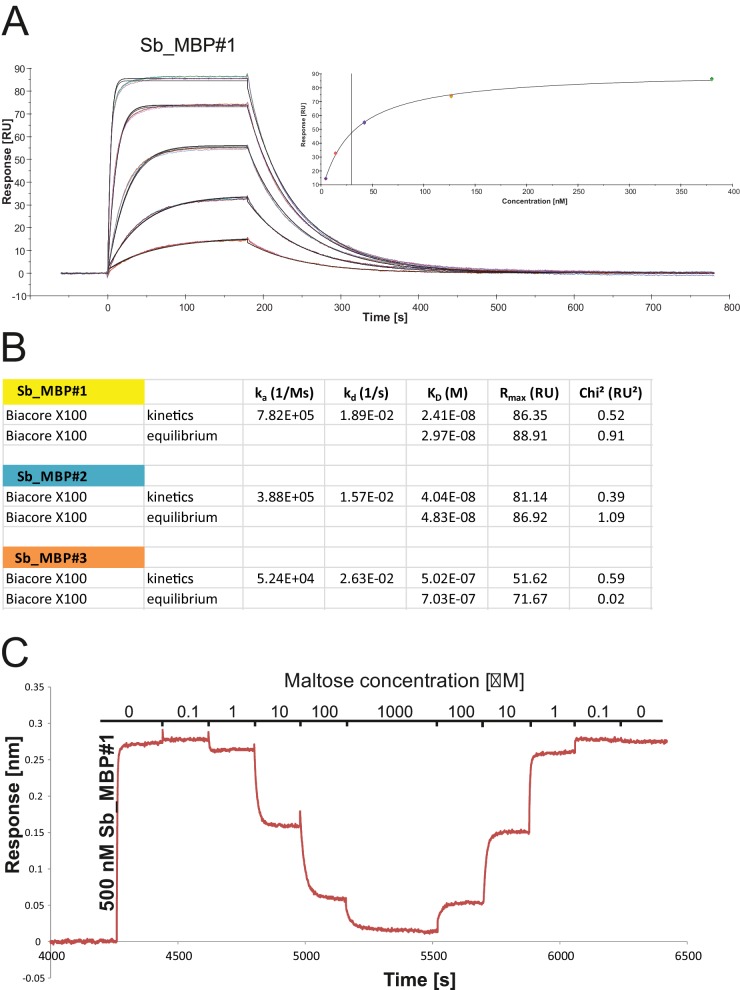
Biophysical analysis of sybody-MBP interactions. (**A**) SPR analysis of interaction between sybody Sb_MBP#1 (analyte) and biotinylated MBP (ligand), determined as technical triplicates for each analyte concentration. Concentrations of Sb_MBP#1: 0, 4.7, 14.1, 42.2, 126.7, 380 nM. Data were fitted with a 1:1 binding model to obtain *k*_on_, *k*_off_ and *K*_D, kinetics_. Inset shows binding equilibrium data to determine *K*_D, equilibrium_. Sybodies Sb_MBP#2 and Sb_MBP#3 were analyzed accordingly. (**B**) Data table summarizing the values obtained from SPR analysis shown in (**A**). (**C**) Displacement of 500 nM Sb_MBP#1 bound to immobilized MBP by addition of increasing maltose concentrations was monitored using the Octet RED96 System.

**Table 3.** FX cloning vectors for phage display and sybody production

Vector name	Description	Resistance marker	Addgene ID
pDX_init	*E.coli* entry and expression vector for FX cloning system, N-terminal PelB signal sequence and C-terminal fusion to PIII for phage display using M13 phages. Nanobodies and sybodies are inserted and excised using SapI or BspQI.	Amp	#110101
pSb_init	*E.coli* entry and expression vector for FX cloning system, N-terminal PelB signal sequence and C-terminal Myc- and 6xHis-tag. Nanobodies and sybodies are inserted and excised using SapI or BspQI.	Cm	#110100
pBXNPH3	*E. coli* expression vector for FX cloning system, N-terminal PelB signal sequence followed by 10xHisTag and 3C cleavage site. Nanobodies and sybodies are inserted using SapI or BspQI.	Amp	#110098
pBXNPHM3	*E.coli* expression vector for FX cloning system, N-terminal PelB signal sequence followed by 10xHisTag, maltose binding protein and 3C cleavage site	Amp	#110099
SB_concave	pBXNPHM3 containing non-randomized framework sybody of the concave library	Amp	#110102
SB_loop	pBXNPHM3 containing non-randomized framework sybody of the loop library	Amp	#110103
SB_convex	pBXNPHM3 containing non-randomized framework sybody of the convex library	Amp	#110104

**Table 4.** Data collection and refinement statistics

	Sb_MBP#1 (PDB: 5M13)	Sb_MBP#2 (PDB: 5M14)	Sb_MBP#3 (PDB: 5M15)
Data Collection			
Space group	P2_1_2_1_2_1_ (19)	P2_1_2_1_2_1_ (19)	P2_1_2_1_2_1_ (19)
Cell dimensions			
a, b, c (Å)	58.298 82.789 102.583	57.890 57.950 281.540	57.030 57.780 286.530
α, β, γ (°)	90.00 90.00 90.00	90.00 90.00 90.00	90.00 90.00 90.00
Resolution (Å)	50–1.37	50–1.6	50–1.9
R_meas_ (%) ^1)^	6.5 (60.9)	5.9 (124)	7.8 (146.6)
*I*/σ*_I_*	15.26 (3.47)	16.98 (1.82)	21.44 (2.17)
CC_1/2_ (%)	99.9 (86.3)	99.9 (68.5)	100 (60.5)
Completeness (%)	99.4 (97.7)	100 (100)	100 (100)
Redundancy	6.1	6.5	12.9
Refinement			
Resolution (Å)	50–1.37	50–1.6	50–1.9
No. reflections (work/test)	102618/5131	126118/6307	75931/3797
*R*_work/_*R*_free_ (%)	16.82/18.60	19.04/21.56	20.92/25.70
No. atoms			
Protein	3873	7640	7619
Water	694	1040	422
B-factor (Å^2^)			
Total	20.1	34.4	50.1
R.m.s deviations			
Bond lengths (Å)	0.005	0.003	0.003
Bond angles (°)	0.750	0.591	0.623

1) Values in parentheses are for the last resolution shell.

### The sybody selection cascade to tackle membrane protein targets

Three consecutive rounds of ribosome display (analogous to the successful selections against MBP) were insufficient to obtain sybodies against membrane proteins, as we demonstrated at the example of the ABC transporter TM287/288 (Figure 1—figure supplement 6A). In order to analyze the problem, we used qPCR to quantify the cDNA corresponding to the eluted mRNA after each selection round. We were thus able to follow the enrichment of binders against the target compared to control proteins. Increasing amounts of mRNA were pulled down in subsequent selection rounds, but to a similar extent for the control proteins and the target, thus indicating selection bias. As every display system provides selective advantage for a particular subset of background binders, we hypothesized that alteration of display systems will allow us to eradicate a major source of selection bias. Furthermore, our qPCR analyses revealed, that the output from the initial ribosome display selection round yields 10^6^–5 × 10^6^ different sybodies, which can be used to generate a focused phage display library covering 10^7^–5 × 10^7^ sybodies. A first test selection using one round of ribosome display followed by two rounds of phage display against TM287/288 resulted in moderate binder enrichment and gave rise to only a few positive ELISA hits (Figure 1—figure supplement 6B). In the context of a separate study, we selected sybodies against the heterodimeric ABC transporter IrtAB, which is a homologue of TM287/288 (sequence identity 27%). We consider TM287/288 and IrtAB to represent similar difficulty levels for binder selections, because they exhibit a highly similar shape and thus a similar number of available epitopes. To further suppress accumulation of background binders, solution panning was performed, that is ribosome or phage display particles were first incubated with IrtAB in solution, followed by a target pull-down via streptavidin/neutravidin-coated surfaces. In addition, surface chemistries were altered in every selection round, namely magnetic Dynabeads Myone Streptavidin T1 for ribosome display, neutravidin-coated Maxisorp microtiter plates for the first phage display round and magnetic Dynabeads Myone Streptavidin C1 for the second phage display round. These alterations at the level of target immobilization together with the combination of ribosome and phage display resulted in a favorable selection outcome, as manifested by a high number of positive ELISA hits obtained after sybody selection (Figure 1—figure supplement 6C). However, only 25% of the sequenced ELISA hits were unique, hinting at diversity bottlenecks in the selection protocol. In addition, the strongest binder exhibited a disappointingly low affinity of only 238 nM. We suspected two potential bottlenecks in our selection cascade, namely the (i) PCR amplification of cDNA to recover the output of the initial ribosome display round and (ii) phage infection of *E. coli* to recover the output of the first phage display selection round. To remove the first bottleneck, we used Taq polymerase instead of proof-reading polymerases for cDNA amplification, since the 3’ to 5’ exonuclease activity of proof-reading polymerases degrades single stranded DNA. To examine the second bottleneck, we measured the infection rate of the M13 phages and found that only 2–5% of the eluted phages after the first panning round were infecting cells, which resulted in a substantial loss of sybody diversity. To compensate for low infection rates, we increased the volume in the first round of phage display from 100 µl to 4.8 ml. When the combined improvements were applied in a selection against TM287/288, excellent enrichment, high number of positive ELISA hits and high affinities down to the single digit nanomolar range were obtained (Figure 1—figure supplement 6D).

In its final form, the sybody platform is built as a selection cascade, starting with one round of ribosome display, followed by two rounds of phage display (Figure 1). Furthermore, immobilization surface chemistries are changed in every selection round. Our selection cascade thus introduces maximal changes in each selection round at the level of binder display and target immobilization and proved highly effective in enriching sybodies against challenging membrane proteins as shown below.

### Conformational trapping of a bacterial ABC transporter

**Figure 3.**
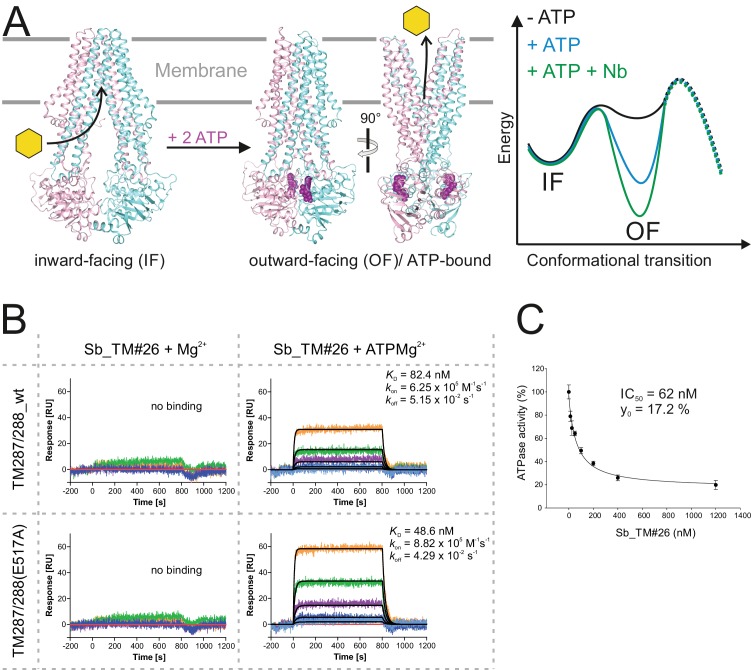
Conformational trapping of ABC transporter TM287/288. (**A**) In the absence of nucleotides, ABC transporter TM287/288 adopts its inward-facing (IF) state and captures substrates from the cytoplasm. ATP binding is required to achieve a partial population of the outward-facing (OF) state, which allows for substrate exit to the cell exterior. Sybodies were selected in the presence of ATP against the transporter mutant TM287/288(E517A), which is incapable of ATP hydrolysis and predominantly populates the OF state in this condition. (**B**) SPR analysis of loop sybody Sb_TM#26 in the presence and absence of ATP using wildtype TM287/288 and TM287/288(E517A) as ligands. Concentrations of Sb_TM#26: 0, 1, 3, 9, 27, 81 nM. (**C**) ATPase activities of wildtype TM287/288 at increasing concentrations of Sb_TM#26. Error bars report the standard deviation of technical triplicates. IC_50_ corresponds to the sybody concentration required for half-maximal inhibition and y_0_ to the residual ATPase activity at saturating sybody concentrations.

**Figure 4.**
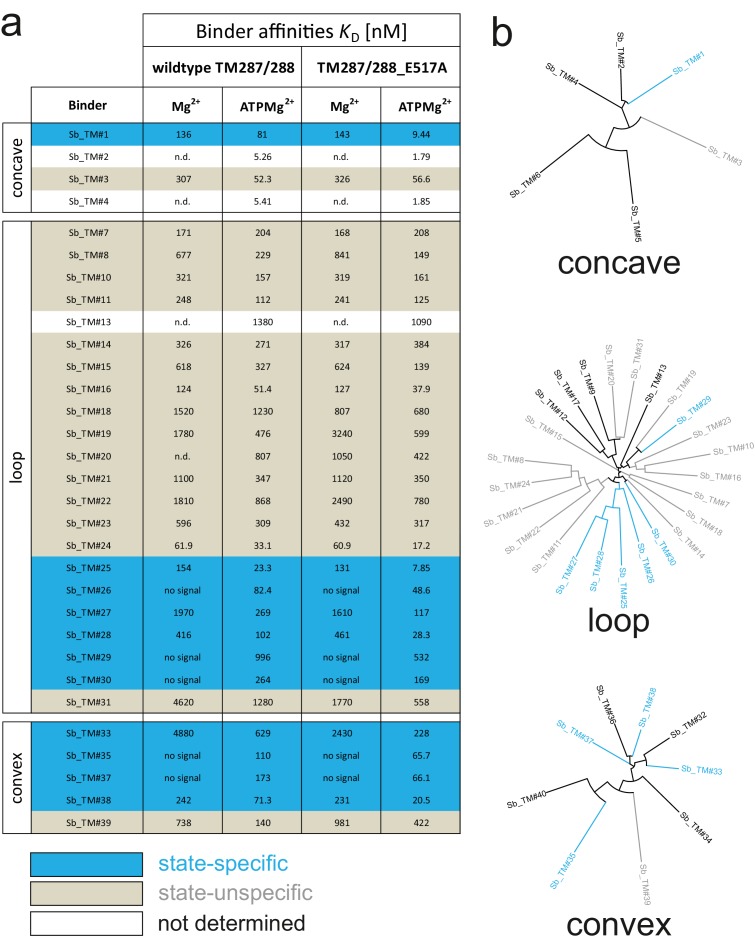
Analysis of sybodies raised against ABC transporter TM287/288. (**A**) Binding affinities of 31 sybodies belonging to the concave, loop and convex library were determined by kinetic SPR measurements using the ProteOn XPR36 Protein Interaction Array System in the presence and absence of ATP and using wildtype TM287/288 and the ATPase-deficient mutant TM287/288(E517A) as ligands. Binders which exhibit an affinity increase of at least ten-fold against TM287/288(E517A) in the presence of ATP were defined as state-specific and are marked in blue. (**B**) Phylogenetic trees of sybodies specific against TM287/288 as determined by ELISA. Note that some of the sybodies were not analyzed by SPR either due to low yields during purification or poor SPR data.

ABC transporters harness the energy of ATP binding and hydrolysis to transport substrates against a concentration gradient. In the absence of ATP, the bacterial ABC transporter TM287/288 almost exclusively adopts an inward-facing (IF) state, and two crystal structures were solved in this conformation (Hohl et al., 2012; Hohl et al., 2014). In contrast, a structure of outward-facing (OF) TM287/288 is still missing due to difficulties in stabilizing this alternate conformation. The transition to the OF state requires ATP binding (Figure 3A) (Timachi et al., 2017), but ATP hydrolysis constantly reverts the transporter back to its IF state. In order to populate the OF state, a glutamate to alanine substitution (E517A) in the ATP-binding cassette was introduced, which blocks ATP hydrolysis without impairing ATP binding (Timachi et al., 2017). Using the sybody platform (Figure 1), binders were selected in vitro against TM287/288(E517A) in the presence of ATP (Figure 3). Using qPCR and AcrB as background control (Seeger et al., 2013), we observed strong sybody enrichment of 170, 220 and 25 fold for the concave, loop and convex library, respectively, after the second round of phage display. For each library, 190 clones were analyzed for binding against TM287/288(E517A) in the presence of ATP by ELISA, of which 60% were ELISA positive. Of 48 sequenced ELISA hits, 40 were unique, indicating high diversity after binder selection (Figure 4, Figure 4—figure supplements 1–3). The unique sybodies were named Sb_TM#1–40, and 37 thereof could be purified at sufficient yields and quality for further analyses. State specificity of these binders was assessed by SPR using wildtype and E517A mutant of TM287/288 as ligands and the sybodies as analytes in the presence and absence of ATP (Figure 3B). SPR data of 31 binders could be quantified and revealed 11 sybodies specific for OF TM287/288(E517A) as defined by an affinity increase of at least ten-fold upon addition of ATP (Figure 4). Sb_TM#26 exhibited a particularly strong state-specificity as binding was exclusively detected in the presence of ATP. Therefore, this binder was analyzed for its capacity to inhibit ATP hydrolysis of TM287/288, revealing an IC_50_ of ATP hydrolysis of 62 nM (Figure 3C). In summary, sybody selections against OF TM287/288 resulted in a large number of high affinity binders trapping the transporter in its ATP-bound state and thereby strongly inhibiting ATPase activities.

**Figure 4—figure supplement 1.**
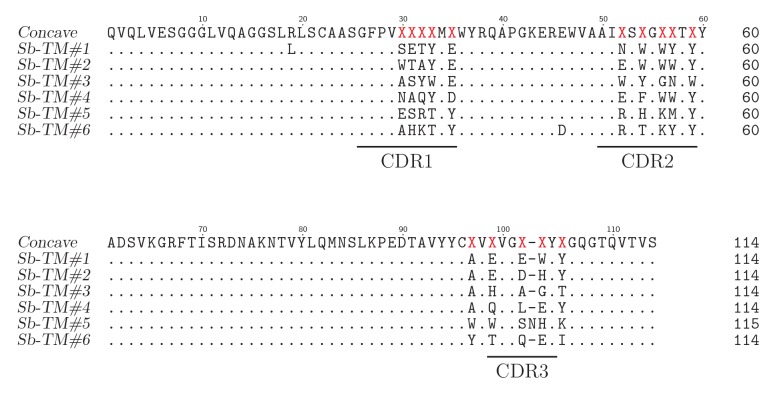
Sequence alignment of concave sybodies raised against TM287/288.

**Figure 4—figure supplement 2.**
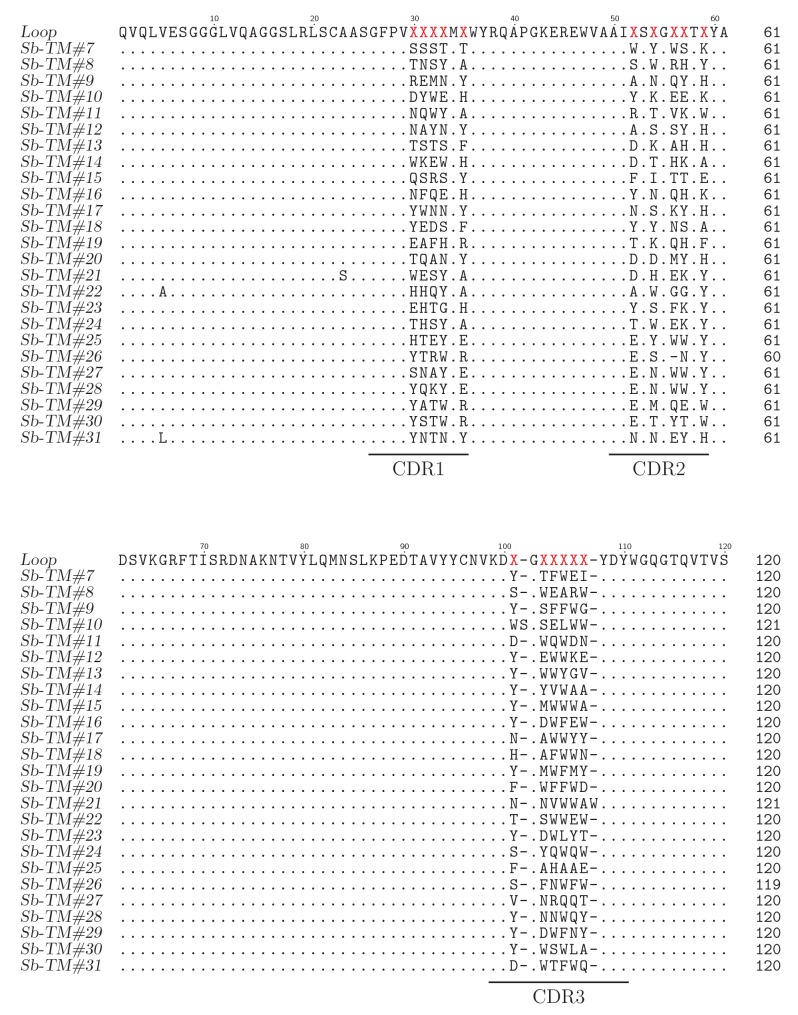
Sequence alignment of loop sybodies raised against TM287/288.

**Figure 4—figure supplement 3.**
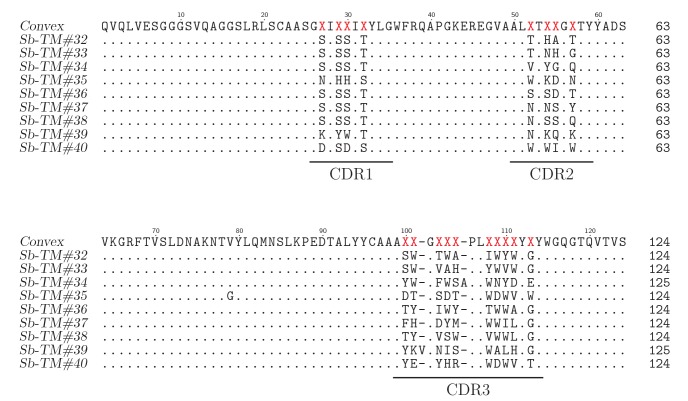
Sequence alignment of convex sybodies raised against TM287/288.

### Conformation-specific stabilization of the human SLC transporters GlyT1 and ENT1

**Figure 5.**
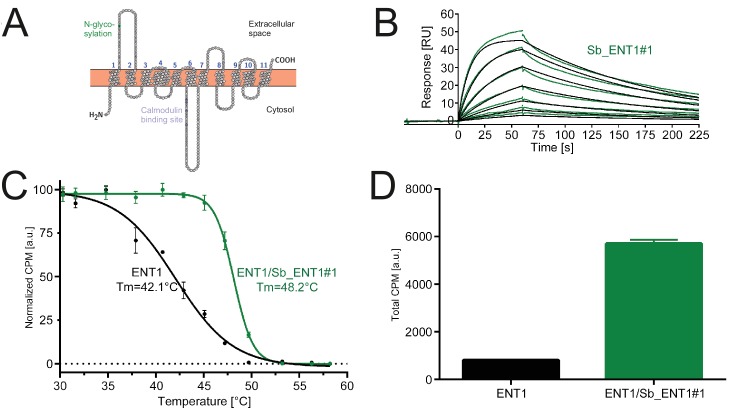
Conformation-specific binding of Sb_ENT1#1 to the inhibition state of human ENT1. (**A**) Snake plot of human ENT1. (**B**) SPR analysis of Sb_ENT1#1 binding to biotinylated ENT1 revealing a *K*_D_ of 40 nM. (**C**) Scintillation proximity assay thermal shift (SPA-TS) analysis of human ENT1 in the presence and absence of Sb_ENT1#1 using [^3^H]-NBTI inhibitor. Error bars correspond to standard deviations of technical triplicates. Sb_ENT1#1 stabilizes an inhibited conformation as evidenced by a shift of the apparent melting temperature (*T_m_*) by 6.1°C and (**D**) a 7-fold increase of the absolute SPA signal measured at 30.1°C.

**Figure 6.**
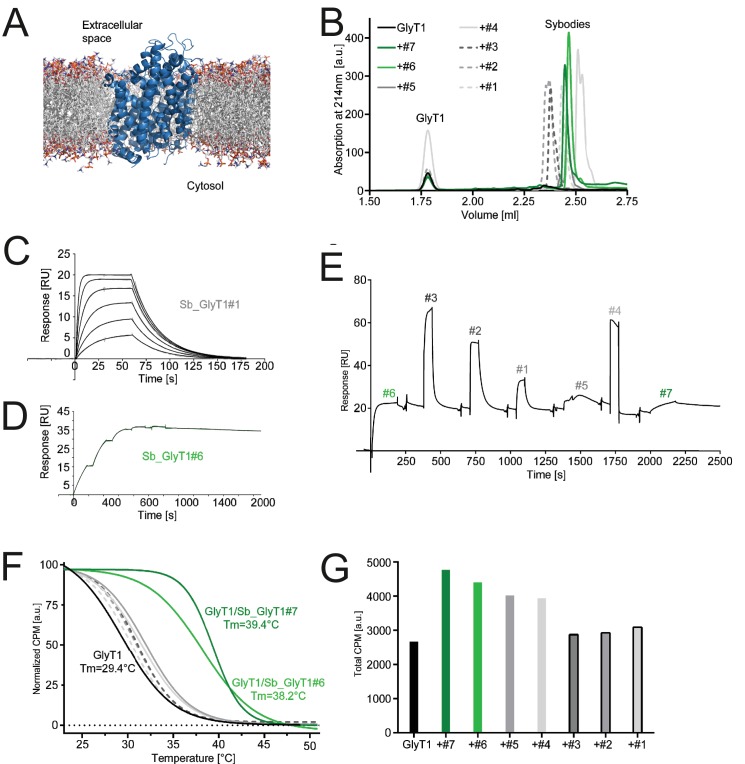
Inhibition-state specific sybodies against human GlyT1. (**A**) Schematic of a GlyT1 homolog (PDP ID: 4M48) embedded in a lipid bilayer, illustrating the limited number of surface-accessible epitopes. (**B**) RP8-HPLC analysis of sybody-GlyT complexes previously separated by SEC. (**C, D**) SPR analysis of Sb_GlyT1#1 (*K*_D_ = 307 nM) and Sb_GlyT1#6 (*K*_D_ = 494 pM). Due to a slow off-rate, SPR analysis of Sb_GlyT1#6 was performed in a single cycle measurement. (**E**) SPR analysis reveals binding of Sb_GlyT1#1–4 to the GlyT1/Sb_GlyT1#6 complex, indicating the presence of two binding epitopes. Sb_GlyT1#5 and Sb_GlyT1#7 compete for binding with Sb_GlyT1#6. (**F**) SPA-TS analysis of Sb_GlyT1#1–7 using [^3^H]-Org24598 reuptake inhibitor. Shifts of the melting temperature (*T*_m_) are highest for Sb_GlyT1#6 and Sb_GlyT1#7 with values of 8.8 and 10°C, respectively, and correlate well with (**G**) increased absolute SPA signals measured at 19°C.

There are only a small number of approved drugs or drugs in development, which therapeutically target human SLC transporters, indicating untapped potential (Lin et al., 2015). A main reason behind these shortcomings is the intricate architecture and low thermal stability that makes human SLC transporters notoriously difficult to work with in early drug discovery stages (César-Razquin et al., 2015). Here we focus on two transporters with a high need for conformation-specific binders for the screening of small molecule therapeutics, namely the equilibrative nucleoside transporter 1 (ENT1, SLC29A1) that is involved in ischemia and acts as a biomarker in pancreatic cancer (Yang and Leung, 2015), as well as on the glycine transporter 1 (GlyT1, SLC6A9) that plays an important role in diseases of the central and peripheral nervous system (Harvey and Yee, 2013) (Figure 5A, Figure 6A). Multiple attempts to raise mouse antibodies or nanobodies against these targets by immunizations failed in our hands, presumably due to low thermal target stability and the limited number of accessible epitopes.

**Figure 5—figure supplement 1.**
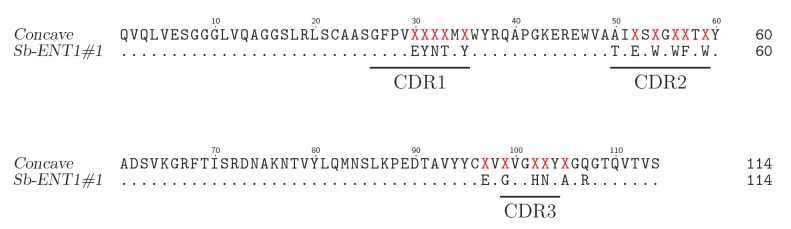
Sequence of Sb_ENT1#1.

**Figure 6—figure supplement 1.**
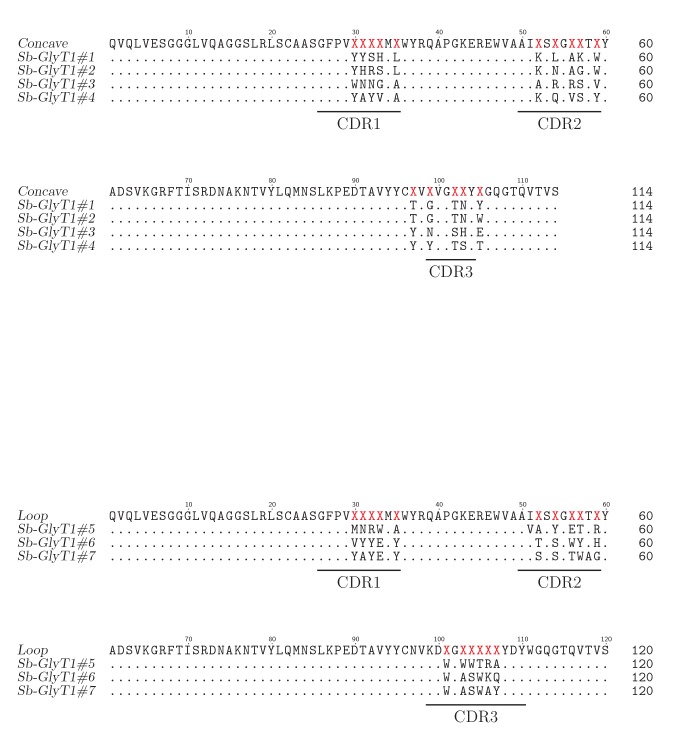
Sequence alignment of sybodies raised against GlyT1.

**Figure 6—figure supplement 2.**
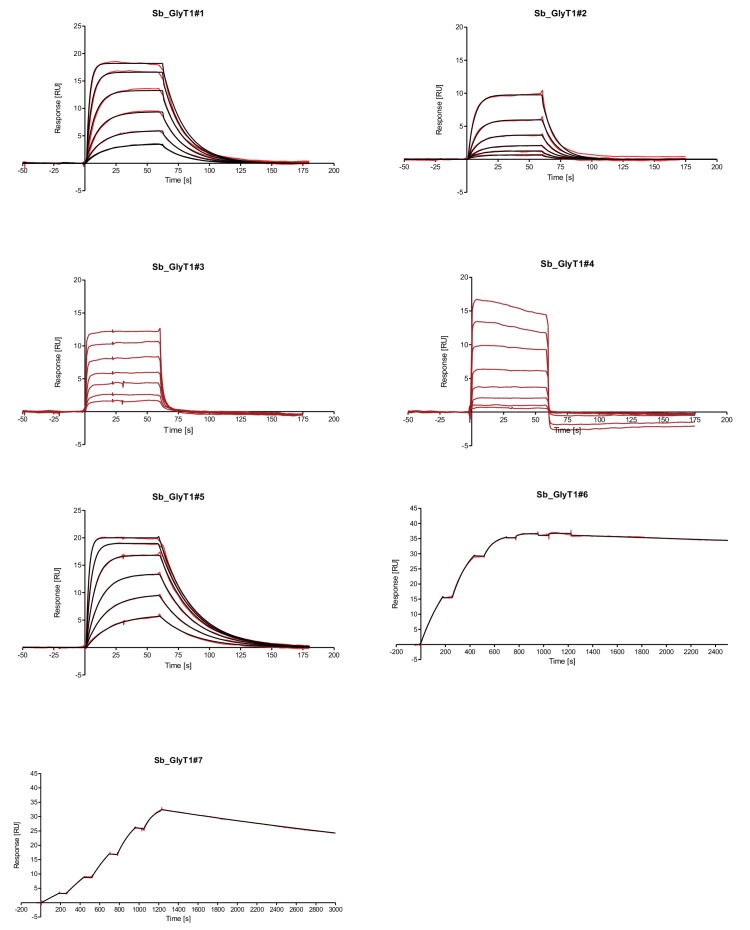
SPR analysis of sybodies raised against ENT1 and GlyT1. Data were fitted using a 1:1 binding model. Representative data of replicates measured on two different SPR chips are shown.

In order to obtain conformation-specific binders against ENT1 and GlyT1, we performed selections at 4°C in the presence of the inhibitors S-(4-Nitrobenzyl)−6-thioinosine (NBTI) and a Bitopertin-like molecule named Cmpd1, respectively. For ENT1, the concave but not the convex and loop library was enriched 4-fold over background after binder selection. One concave sybody called Sb_ENT1#1 was identified by ELISA and purified as monodisperse protein (Figure 5—figure supplement 1). SPR measurements revealed an affinity of 40 nM (Figure 5B, Table 5). To further characterize Sb_ENT1#1, a thermal shift scintillation proximity assay (SPA-TS) was established. ENT1 in complex with the sybody was incubated at varying temperatures in the absence of inhibitor, followed by measuring binding of tritiated NBTI. Sb_ENT1#1 binding led to a sharper transition trajectory and an increase in melting temperature by 6.1°C (Figure 5C), indicating that sybody binding increases the population of the inhibited conformation of ENT1. Supporting this notion, the absolute binding signal for NBTI was increased by more than seven-fold in the presence of Sb_ENT1#1 at temperatures well below *T*_m_ (Figure 5D).

**Table 5.** Characterization of sybodies raised against ENT1 and GlyT1.

Sybody	k_on_ [M^−1^S^−1^]	k_off_ [s^−1^]	K_D_ [M] (kinetics)	K_D_ [M] (equilibrium)	ΔT_m_(SPA-TS) [°C]	SPA signal (fold increase)
Sb ENT1#1	1.86E+05	7.44E-03	4.00E-08		6.1	7
Sb_GlyT1#1	1.88E+05	5.77E-02	3.07E-07		0.9	1.2
Sb_GlyT1#2*	3.68E+04	9.28E-02	2.52E-06		1.5	1.1
Sb_GlyT1#3**				1.54E-07	1.7	1.1
Sb_GlyT1#4**				4.761E-07	2.1	1.5
Sb_GlyT1#5	4.54E+05	3.72E-02	8.19E-08		2.6	1.5
Sb_GlyT1#6***	1.00E+05	4.99E-05	4.94E-10		8.8	1.6
Sb GlyT1#7***	2.01E+04	1.85E-04	9.18E-09		10	1.8

For GlyT1, seven sybodies from the concave (Sb_GlyT1#1–4) and loop (Sb_GlyT1#5–7) library were identified by ELISA and complex formation was confirmed by size-exclusion chromatography (SEC) (Figure 6B, Figure 6—figure supplement 1). Binding kinetics were determined by SPR revealing a wide range of affinities from 494 pM to 2.52 µM (Figure 6C and D, Table 5, Figure 6—figure supplement 2). Competition binding SPR analysis using GlyT1 pre-saturated with Sb_GlyT1#6 revealed binding of concave sybodies Sb_GlyT1#1–4 but not of loop sybodies Sb_GlyT1#5 and Sb_GlyT1#7 (Figure 6E), demonstrating that at least two non-overlapping epitopes on GlyT1 are recognized. The large differences in affinities correlated well with SPA-TS analysis using the commercially available tritiated inhibitor Org24598 that addresses the same binding site as Cmpd1 (Alberati et al., 2012). Of the seven sybodies, Sb_GlyT1#1–5 increased the *T*_m_ by 0.9–2.6°C, whereas Sb_GlyT1#6 and Sb_GlyT1#7 stabilized the transporter by 8.8 and 10°C, respectively (Figure 6F). Absolute SPA binding signals obtained at 19°C increased up to 1.8-fold (Sb_GlyT1#7), suggesting that all sybodies stabilize the inhibited conformation of GlyT1 (Figure 6G).

In conclusion, our selection at 4°C and in the presence of non-covalent inhibitors enabled the rapid identification of sybodies against instable and previously intractable human membrane proteins. The identified binders trap inhibited conformations of ENT1 and GlyT1 and thereby enhance ligand binding. Hence, these binders increase assay sensitivity for inhibitor screening and can serve as crystallization chaperones for structure-based drug design.

## Discussion

Binders are enabling tools to investigate membrane proteins and to stabilize these inherently flexible machineries in defined conformational states for X-ray crystallography as well as single particle cryo-EM. However, selecting conformation-specific binders against membrane proteins has so far been difficult, laborious and not readily accessible to every lab. Here, we developed a robust, fast and inexpensive open-access platform, which entirely operates in vitro and does not require access to animal facilities. Thereby, we operate independent of target toxicity and sequence conservation and allow for a wide range of selection conditions including low-affine or toxic ligands to trap membrane proteins in desired conformations.

Libraries based on synthetic scaffolds often exhibit a single shape and are randomized at only one region of their surface. Since a large fraction of the membrane protein surface is buried beneath lipids or detergent micelles, suboptimal shape-complementarity between its few accessible epitopes and the randomized binder surface is a key limiting factor that can impede successful selections. To overcome this barrier, we engineered three synthetic single domain antibody libraries of different shapes, a strategy that had been applied previously to monobodies and DARPins (Koide et al., 2012; Schilling et al., 2014). Thereby, we created a large paratope space, which is a key feature of the sybody platform to target membrane proteins with a limited number of suitable epitopes.

A thorough investigation of published nanobody structures revealed that each nanobody contains a dedicated set of aromatic or aliphatic CDR residues, which point towards its hydrophobic core and thereby contribute to scaffold stability. Importantly, scaffolding CDR residues are harmonized among CDRs of the same nanobody (see for example 1ZVH and convex library), but vary among different camelid nanobodies. Consequently, CDRs of nanobodies cannot be exchanged without the risk of destabilizing the scaffold. We took this into account by engineering the three sybody scaffolds based on individual nanobody structures. Thereby, we achieved high thermal stability of the sybodies. Structure-based scaffold designs had been successfully applied in the past to construct Fab libraries based on the highly stable humanized Fab-4D5 fragment (Fellouse et al., 2004), monobody libraries on the tenth FN3 unit of human fibronectin (Koide et al., 1998) and anticalin libraries based on the human lipocalin protein Lcn2 (Schönfeld et al., 2009).

Besides the favorable biophysical library properties, large binder diversities are critical for in vitro selections, in order to compensate for affinity maturation taking place in animals as a result of somatic hypermutation. In this work, we show that nanobodies and sybodies can be efficiently displayed on ribosomes using a commercial kit. Thereby, 10^12^ binder candidates can be displayed with minimal effort (Zahnd et al., 2007), whereas phage or yeast display libraries are typically limited to 10^8^–10^10^ library members (McMahon et al., 2018; Moutel et al., 2016; Yan et al., 2014). By systematically monitoring output cDNA amounts using qPCR, we learned that the initial ribosome display round generates a sybody pool with a maximal diversity of 5 × 10^6^, from which a focused phage display library can be constructed with moderate effort in analogy to nanobody cloning from B-cells of immunized camelids (Pardon et al., 2014). We further realized that binder generation against challenging membrane proteins benefits from radical changes in display format and target immobilization between each selection round to overcome otherwise inevitable biases. The sybody platform is therefore built as a selection cascade, in which the library is pre-enriched by ribosome display and then funneled into a phage display library of optimal size.

In recent years, the generation of three synthetic nanobody libraries has been described (McMahon et al., 2018; Moutel et al., 2016; Yan et al., 2014). For clarity of discussion, these synthetic nanobody libraries are henceforth called McMahon, Moutel and Yan. The Moutel and McMahon libraries were constructed according to a consensus design approach, either based on stable natural nanobodies (Moutel et al., 2016) or a large collection of PDB entries (McMahon et al., 2018). The CDR3s of the Moutel library exhibit lengths of 9, 12, 15 and 18 aa. The Yan library is only randomized in CDR3 with a fixed length of 16 aa. The CDR3 lengths of the McMahon library are 10, 14 and 18 aa according to the standard counting system (Sircar et al., 2011), but were counted as 7, 11 and 15 aa by the authors (McMahon et al., 2018). Shorter binder variants of the McMahon and Moutel libraries (CDR3s ranging from 9 to 14 aa) are comparable in terms of shape and randomized surface to our loop sybodies (CDR3 of 12 aa). However, longer variants of the McMahon and Moutel libraries (CDR3s ranging from 15 to 18 aa) as well as the Yan library (CDR3 of 16 aa) do not contain an engineered hydrophobic core to tether the long CDR3s, as it was designed for the convex library with its 16 aa CDR3 based on its template nanobody structure 1ZVH. Importantly, natural nanobodies from camelids with CDR3 loops ≥ 16 aa always feature an extended hydrophobic core to tether CDR3 (Sircar et al., 2011). In addition, many of them contain a second disulfide bond to restrict the flexibility of the long CDR3 loops (Sircar et al., 2011). Hence, our convex sybody library is currently the only synthetic nanobody library with a long CDR3 that mimics loop tethering as found in camelid nanobodies.

For a direct comparison of the binding modes of the different synthetic nanobody libraries, many structures in complex with the respective targets would be required, but are unfortunately not available in large numbers. The most recent study contains a structure of a synthetic nanobody with a medium-sized CDR3 loop (10 aa) in complex with human serum albumin (McMahon et al., 2018). Although not intended by library design, the synthetic nanobody binds side-ways via a concave interaction surface akin to the GFP nanobody 3K1K (Kirchhofer et al., 2010). In contrast, the structures of our convex sybody/MBP complexes revealed binding via the designed convex surfaces into a cleft of the target protein, thus confirming the anticipated binding mode.

To explore the robustness and the potential of our platform, we generated state-specific sybodies against the soluble protein MBP, one bacterial ABC transporter and two human SLC transporters. MBP was chosen as a simple test case to validate our sybody libraries, because it is very stable and was previously used to validate synthetic binder libraries (Binz et al., 2004; Gilbreth et al., 2008; Rizk et al., 2011). A large set of diverse sybodies of all three libraries exhibiting affinities down to 0.5 nM was readily obtained. X-ray structures of three closely related convex sybodies in complex with MBP proved the integrity of the sybody scaffold as well as the utility of the randomized binding surface.

In a second step, we generated sybodies that specifically recognize the transient ATP-bound state of the bacterial ABC transporter TM287/288. This state cannot be populated in an animal during immunization, because ATP dissociates after target injection. Despite of the fact that TM287/288 is an integral membrane protein, we considered it as a target of intermediate difficulty, because it is stable and contains hydrophilic nucleotide binding domains providing a large epitope space. Nevertheless, an improved selection protocol combining ribosome and phage display was required to obtain binders against this ABC transporter. A high percentage of the identified sybodies were found to be state-specific, indicating that our in vitro selection process in the presence of ATP efficiently enriched binders against the ATP-bound state of TM287/288.

The most remarkable achievement of our platform was the rapid generation of conformation-selective sybodies against the disease-relevant drug discovery transporters ENT1 and GlyT1. These human SLC transporters unite all attributes of very challenging membrane protein targets: They are intrinsically flexible, heat labile and contain only a very limited hydrophilic surface that can be addressed by binders. It is therefore not surprising that we were initially unable to generate antibodies or nanobodies via animal immunization against these two targets. However, using our selection cascade and performing selections at 4°C in the presence of stabilizing inhibitors, we identified a handful of sybodies of the convex and loop libraries exhibiting favorable biophysical properties and high affinities down to the picomolar range. As expected, enrichment and the number of binders identified were considerably lower than for the less challenging bacterial ABC transporter. Importantly, using a newly established thermal shift scintillation proximity assay (SPA-TS), we could demonstrate that the identified sybodies lock the transporters in their inhibitor-bound conformation and thereby increased the thermal stability of these SLC transporters by up to 10°C.

Within the selections against these four targets, no winning scaffold has emerged. While members of all three libraries were identified for MBP and the ABC transporter TM287/288, the concave and the loop library gave better results for the two human SLCs, potentially reflecting the limited number of accessible epitopes, which might only be complementary to a particular subset of binder shapes. Therefore, we recommend using all three sybody libraries for future targets. In order to facilitate the spread and further development of the technology, the libraries will be made fully available to academic labs and the phage display and sybody expression vectors were made available through Addgene. In conclusion, the sybody platform is a remarkably fast and reliable technology enabling the next generation of challenging drug discovery targets including receptors, channels and transporters.

## Materials and methods

### Construction of vectors for phage display and sybody expression

An FX cloning vector for the periplasmic production of VHHs preceded by an N-terminal decaHis-tag and an HRV 3C protease cleavage side, designated pBXNPH3 (Figure 1—figure supplement 5), was constructed by polymerase chain reaction (PCR) using Phusion polymerase and pBXNH3 (Geertsma and Dutzler, 2011) as template in combination with the 5’-phosphorylated primers pBXNPH3_#1 and pBXNPH3_#2. The resulting 5848 bp product was DpnI-digested, column-purified, ligated and transformed to chemically-competent *ccdB*-resistant *E. coli* DB3.1. A variant designated pBXNPHM3 (Figure 1—figure supplement 5), which is compatible with the periplasmic production of VHHs as a fusion to an N-terminal decaHis-tag, maltose binding protein (MBP) and HRV 3C protease site, was constructed from fragments of pBXNH3 (Geertsma and Dutzler, 2011) amplified using primer pair pBXNPHM3_#1 (holding an NotI restriction site) and pBXNPHM3_#2 (5’-phosphorylated) and pET26FX (Bertozzi et al., 2016) amplified using primer pair pBXNPHM3_#3 (5’-phosphorylated) and pBXNPHM3_#4 (holding an NotI restriction site). The resulting products of 4241 bp (vector backbone) and 2732 bp (insert holding *mbp* and *ccdB*) bp, respectively were DpnI-digested, gel-purified, cut with NotI, ligated and transformed into *E. coli* DB3.1. To obtain pSb_init, the ampicillin (*amp*) resistance gene of pBXNPH3 was replaced by a chloramphenicol (*cat*) marker. Because the kill cassette of pBXNPH3 contained a chloramphenicol marker as well, pBXNPH3 containing nanobody 1ZVH was used as template to amplify the vector without the *amp* gene with the primer pair pBXNPH3_blunt_for/pBXNPH3_EcoRI_rev. The *cat* gene was amplified from pINIT_cat (Geertsma and Dutzler, 2011) (Addgene #46858) using primers Cm_EcoRI_for and Cm_blunt_rev (5’-phosphorylated). The PCR products were column purified, digested with EcoRI, purified by gel extraction, ligated and transformed into *E. coli* MC1061. The resulting plasmid was amplified by primers Nb_init_for (5’ phosphorylated) and Nb_init_rev and circularized by ligation. The resulting vector was cut with SapI and the kill cassette, excised from pINIT_cat using the same restriction enzyme, was inserted resulting in vector pSb_init. The FX cloning vector for phage display, named pDX_init, was constructed based on pMESy4 (Pardon et al., 2014). The internal SapI site preceding the lac promoter in pMESy4 was removed by generating a short 500 bp PCR fragment with a mutation in the SapI recognition site using the primer pair pDX_init_#1/pDX_init_#2. The NcoI/SapI-digested PCR product was gel-purified and ligated into NcoI/SapI-digested and dephosphorylated pMESy4 and transformed into *E. coli* MC1061. The amber stop codon on the resulting vector was replaced by a glutamine residue using Quickchange mutagenesis using the primer pair pDX_init_#3/pDX_init_#4. The resulting vector backbone was amplified using primer pair pDX_init_#5/pDX_init_#6, thereby introducing SapI sites as part of the open reading frame, digested with SapI and ligated with a SapI-digested PCR-fragment holding the counterselection marker *sacB* amplified from pINIT (Geertsma and Dutzler, 2011) using primer pair pDX_init_#7/pDX_init_#8.

### Sybody expression and purification

Sybodies were expressed in *E. coli MC1061*, which were grown in terrific broth containing 25 µg/ml chloramphenicol (in case of pSb_init) or 100 µg/ml ampicillin (in case of pBXNPH3 as well as pBXNPHM3) to an OD_600_ of 0.7 at 37°C. Then the temperature was lowered to 22°C and cells were induced with 0.02% (w/v) L-arabinose for 15 hr. Cells were disrupted using a microfluidizer processor (Microfluidics, Westwood, MA, United States) at 25’000 lb/in^2^ in TBS (20 mM Tris-HCl pH 7.5, 150 mM NaCl) supplement with 2 mM MgCl_2_ and 25 μg/ml DNAse I. Cell debris was removed by centrifugation at 8’000 g for 20 min and 15 mM imidazole pH 7.5 was added prior to loading onto a gravity flow Ni-NTA superflow column of 2 ml bed volume (Qiagen, Venlo, The Netherlands). The column was washed with 25 ml TBS containing 30 mM imidazole pH 7.5 in case of pSb_init (50 mM in case of pBXNPH3 or pBXNPHM3) and the sybody was eluted with 5 ml TBS containing 300 mM imidazole pH 7.5. If expressed in pBXNPH3 or pBXNPHM3, the Ni-NTA purified sybodies were dialyzed against TBS in the presence of 3C protease for 3 hr and loaded onto Ni-NTA columns to remove the His-tag or the His-tagged MBP as well as the 3C protease. Tag-free sybodies were eluted from the Ni-NTA column using TBS containing 30 mM imidazole. Sybodies were concentrated using centrifugal filters with a 3 kDa cut-off (Amicon Ultra-4) and separated by size exclusion chromatography (SEC) using Superdex 200 Increase 10/300 GL (GE Healthcare, Glattbrugg, Switzerland) or Sepax-SRT10C SEC-300 (Sepax Technologies, Newark, DE, United States) in TBS.

### Assembly of sybody libraries

Synthetic genes encoding for three non-randomized scaffold sybodies (convex, loop and concave) were ordered at DNA2.0 (Table 2). These scaffold sybodies contain serines and threonines in the positions to be randomized in the respective libraries and served as PCR templates for library assembly. Their sequences harbored within expression vector pBXNPHM3 were made available through Addgene (Table 3). Primers (Table 6) were added to the PCR reaction at a concentration of 0.8 µM if not specified differently. Primers for the sybody assembly were ordered in PAGE-purified form (Microsynth, Balgach, Switzerland). Randomized primers were synthetized using trimer phosphoramidites (Ella Biotech, Martinsried, Germany). If not otherwise mentioned, Phusion High-Fidelity DNA Polymerase (NEB, Ipswich, MA, United States) was used for PCR amplification. Five double-stranded PCR products, which served as megaprimers in the assembly of the library were first amplified from the genes encoding for the frameworks of the concave/loop and convex library. The gene of the concave/loop sybody framework was amplified with primer pairs FW1_a_b_for/FW1_a_b_rev (megaprimer 1), FW3_a_b_for/Link2_a_rev (megaprimer 2) or FW3_a_b_for/Link2_b_rev (megaprimer 3). The gene of the convex sybody framework was amplified with primer pairs FW1_c_for/FW1_c_rev (megaprimer 4) and FW3_c_for/Link2_c_rev (megaprimer 5). Megaprimers were gel-purified. In a second step, the individual CDR regions of the libraries were assembled by overlap extension PCR using Vent DNA Polymerase (NEB), applying 35 cycles and an annealing temperature of 60°C. 100 µl of the PCR reactions contained 10 µl 10 x Vent buffer, 1 µl Vent DNA polymerase, 5 µl DMSO, 0.4 mM dNTPs, 1 µM outer primers, 50 nM randomized primer, 25 nM megaprimers (if applicable) and 25 nM internal assembly primer (if applicable). Table 7 lists the primers used for the assembly of the CDRs of the respective libraries. The assembly PCR reactions yielded single DNA species of the expected size, which was purified by PCR purification kit (Qiagen). Fragments containing CDR1 were digested with BsaI and fragments containing CDR2 with BpiI. Digestion with these two Type IIS restriction enzymes resulted in complementary sticky ends of 4 base pairs. Digested DNA was purified by PCR purification kit and CDR1-CDR2 pairs belonging to the respective library were ligated with T4 ligase. Separation of the ligation product by DNA gel revealed almost complete ligation of the fragments. The three ligation products consisting of the respective CDR1-CDR2 pairs of the three libraries were purified by gel extraction and yielded around 800 ng of DNA. The purified ligation product served as template to amplify the CDR1-CDR2 region using primer pairs FW1_a_b_for/Link2_a_rev, FW1_a_b_for/Link2_b_rev and FW1_c_for/Link2_c_rev for the concave, loop and convex library, respectively. The resulting PCR product was cleaned up by PCR purification kit and digested with BsaI. The purified CDR3 regions were digested with BpiI. The resulting compatible overhangs were ligated and the ligation product corresponding to the final assembled library was purified by gel extraction. The DNA yields were 6.8, 8.6 and 9.4 µg for the concave, loop and convex library, respectively.

**Table 6.** List of Primers

Primers for library assembly (triplets designated 111, 222 and 333 correspond to the trinucleotide mixtures 1–3 for randomization; all primers in 5’ to 3’ orientation)	
CDR1_a_b	GCA AGC GGT TTC CCG GTG 111 111 111 222 ATG 333 TGG TAT CGT CAG GCA CCG G
CDR1_c	C TGT GCG GCT AGC GGC 111 ATT 111 111 ATC 222 TAC CTG GGC TGG TTT CGC C
CDR2_a_b	GA AGA CCT GTC GCG GCG ATT 111 AGC 111 GGT 111 222 ACG 333 TAC GCA GAT TCT GTT AAG GGC CG
CDR2_c	CGA AGA CCT GCA GCG CTG 111 ACC 111 111 GGT 222 ACC TAC TAC GCG GAC AGC G
CDR3_a	GA AGA CCT GCG GTT TAC TAC TGT 333 GTG 222 GTG GGT 111 222 TAC 333 GGC CAA GGT ACC CAA GTG AC
CDR3_b	CGC GAA GAC CTC GTG AAA GAC 111 GGT 111 111 111 111 111 TAC GAC TAT TGG GGC CAA GGT ACC CAA GTG AC
CDR3_c	GAA GAC CTC TGC GCG GCA GCC 111 111 GGC 111 111 111 CCG CTG 111 111 111 111 TAT 222 TAC TGG GGT CAG GGC ACC CAA GTT ACC GTT TCT
FW1_a_b_for	CAG GTT CAG CTG GTT GAG AGC
FW1_a_b_rev	CAC CGG GAA ACC GCT TGC
FW1_c_for	CAA GTC CAG CTG GTG GAA TCG
FW1_c_rev	GCC GCT AGC CGC ACA G
FW2_a_b_rev	ATG CAT GGT CTC ACG ACC CAC TCA CGT TCT TTG CCC GGT GCC TGA CGA TAC CA
FW2_c_rev	ATG CAT GGT CTC ACT GCG ACG CCC TCA CGC TCT TTG CCC GGT GCC TGG CGA AAC CAG CCC AGG
FW3_a_b_for	CGC AGA TTC TGT TAA GGG CCG
FW3_c_for	ACC TAC TAC GCG GAC AGC G
FW4_a_b_rev	GCT CAC AGT CAC TTG GGT ACC TTG GCC
FW4_c_rev	AGA AAC GGT AAC TTG GGT GCC CTG
Link1_a_b_for	ATG CAT GAA GAC CTG TCG CGG CG
Link1_a_b_rev	ATG CAT GGT CTC ACG ACC CAC
Link1_c_for	TAT ATC GAA GAC CTG CAG CGC TG
Link1_c_rev	ATG CAT GGT CTC ACT GCG ACG
Link2_a_for	TAT ATC GAA GAC CTG CGG TTT ACT ACT G
Link2_a_rev	ATG CAT GGT CTC ACC GCG GTA TCT TCC GGT TTC
Link2_b_for	ATG CAT GGT CTC ACC GCG GTA TCT TCC GGT TTC
Link2_b_rev	ATG CAT GGT CTC ACA CGT TAC AGT AGT AAA CCG CGG
Link2_c_for	ATA TAT GAA GAC CTC TGC GCG GC
Link2_c_rev	ATG CAT GGT CTC AGC AGT AAT ACA AAG CAG TAT CTT CCG G
Primers for vector construction	
pBXNPH3_#1	CAG CAG TCC GGC AGC AGC GGT CGG CAG CAG GTA TTT CAT GGT TAA TTC CTC CTG TTA GCC
pBXNPH3_#2	CTC CTC GCT GCC CAG CCT GCA ATG GCC GCA GAT CAC CAT CAT CAT CAC CAT CAT CAT CAT CAT TTA
pBXNPHM3_#1	ATA TAT GCG GCC GCC ATA GTG ACT GGA TAT GTT G
pBXNPHM3_#2	CAT GGT TAA TTC CTC CTG TTA GCC CAA AAA
pBXNPHM3_#3	AAA TAC CTG CTG CCG ACC GCT GCT GCT GGT
pBXNPHM3_#4	ATA TAT GCG GCC GCA TTA GGC ACC CCA GGC TTT A
pBXNPH3_blunt_for	CTC ATG ACC AAA ATC CCT TAA CGT GAG
pBXNPH3_EcoRI_rev	ATA TAT GAA TTC ATG GGG AGA CCC CAC ACT AC
pDX_init_#1	ATA TAT GCT CTT CAA GCG GAA GAG AGC CCA ATA CGC AAA CCG
pDX_init_#2	CGT TAG TAA ATG AAT TTT CTG TAT GAG GTT TTG
pDX_init_#3	GAA CCT GAA GCC CAG TAC CCG TAC
pDX_init_#4	CGT ACG GGT ACT GGG CTT CAG GTT
pDX_init_#5	TAT AAC TTG AAG AGC CGG CTG CCA TGG CCG GCT GGG CC
pDX_init_#6	TAT AGC AGG AAG AGC TCA CCA CCA TCA CCA TCA CGA ACC TG
pDX_init_#7	TAT AGC TCT TCA AGT CTG CCC ACA TAT ACC TGC CGT TC
pDX_init_#8	TAT AGC TCT TCC TGC AGA CAC GTG TCA CGT GAG GCC
Cm_EcoRI_for	GCT CAT GAA TTC CCC GCG CG
Cm_blunt_rev	GTG CAA TGT AAC ATC AGA GAT TTT GAG ACA C
Nb_init_for	ATG CAG GAA GAG CTG GCG AAC AAA AAC TCA TCT CAG AAG AGG ATC TG
Nb_init_rev	ATA CTT GAA GAG CCG GCC ATT GCA GGC TGG GCA G
RD_FX_pRDV_for	ATA TAT GCT CTT CTG CAA AGC TTT ATA TGG CCT CGG GGG C
RD_FX_pRDV_rev1	TAT ATA GCT CTT CAA CTA CCC ATG GAT ATA TCT CCT TCT TAA AGT TAA AC
pRDV_SL_for	AGA CCA CAA CGG TTT CCC TCT AGA AAT AAT TTT GTT TAA CTT TAA G
pRDV_SL_rev	CCC TAT AGT GAG TCG TAT TAA TTT CGA TGG
GS-Linker_FW	GGC GGT GGC GGT AGT AGA AGA GCG AGC TGC AGA CTG
GS-Linker_RV	GCC GGA ACC ACT TGG ACC TTG AAA CAA AAC TTC TAA ATG ATG
Primers for target amplification	
GFP_FX_FW	TAT AGC TCT TCT AGT CAA TTC AGC AAA GGA GAA GAA CTT TTC
GFP_FX_RV	TAT AGC TCT TCT TGC TGC ACT AGT TTT GTA GAG CTC ATC C
MBP_FX_FW	ATA TAT GCT CTT CTA GTA AAA TCG AAG AAG GTA AAC TGG TAA TCT GG
MBP_FX_RV	TAT ATA GCT CTT CAT GCG CTA CCC GGA GTC TGC GC
IrtAB_FX_FW	ATA TAT GCT CTT CTA GTC TTC GTG GAC TGG GTG CCC GCG ACC AT
IrtAB_FX_RV	TAT ATA GCT CTT CAT GCC CGT GCC GTC GAC CCG ATC GCC CAC TC
Primers for ribosome and phage display	
Med_FX_for	ATA TGC TCT TCT AGT CAG GTT CAG CTG GTT GAG AGC G
Med_FX_rev	TAT AGC TCT TCA TGC GCT CAC AGT CAC TTG GGT ACC
Long_FX_for	ATA TGC TCT TCT AGT CAA GTC CAG CTG GTG GAA TCG
Long_FX_rev	TAT AGC TCT TCA TGC AGA AAC GGT AAC TTG GGT GCC C
RT_Primer	CTT CAG TTG CCG CTT TCT TTC TTG
Medium_ORF_for	AGT CAG GTT CAG CTG GTT GAG AGC G
Medium_ORF_rev	TGC GCT CAC AGT CAC TTG GGT ACC
Long_ORF_for	AGT CAA GTC CAG CTG GTG GAA TCG
Long_ORF_rev	TGC AGA AAC GGT AAC TTG GGT GCC C
5'_flank _for	CGA AAT TAA TAC GAC TCA CTA TAG GGA GAC
tolAk_rev	CCG CAC ACC AGT AAG GTG TGC GGT TTC AGT TGC CGC TTT CTT TCT
tolAk_2	CCG CAC ACC AGT AAG GTG TGC
5'_flank _rev	TAT AGC TCT TCA ACT ACC CAT GGA TAT ATC TCC
3’_flank_for	TAT AGC TCT TCT GCA AAG CTT TAT ATG GCC TC
Medium_ORF_5'_rev	CGC TCT CAA CCA GCT GAA CCT GAC T
Long_ORF_5'_rev	CGA TTC CAC CAG CTG GAC TTG ACT
Medium_ORF_3’_for	GGT ACC CAA GTG ACT GTG AGC GCA
Long_ORF_3'_for	GGG CAC CCA AGT TAC CGT TTC TGC A
Primers for qPCR	
qPCR_RD_5’_for	GGG AGA CCA CAA CGG TTT CCC
qPCR_ RD_S and M_5’_rev	CAC CGG GAA ACC GCT TGC GGC
qPCR_ RD_L_5’_rev	GCC GCT AGC CGC ACA GCT C
qPCR_ RD_tolA_3’_for	GCC GAA TTC GGA TCT GGT GGC
qPCR_ RD_tolA_3’_rev	CTG CTT CTT CCG CAG CTT TAG C
qPCR_PD_pDX_for	GAC GTT CCG GAC TAC GGT TCC
qPCR_PD_pDX_rev	CAC AGA CAG CCC TCA TAG TTA GC
qPCR_3K1K_for	AGT GCC GGT GAT CGT AGC AG
qPCR_3K1K_rev	CCC AAT ATT CAA AGC CCA CGT T

**Table 7.** Primers and megaprimers used to assembly the sybody libraries

Library	CDR	Randomized primer	Megaprimer	Assembly primer	Outer primers
concave	CDR1	CDR1_a_b	Megaprimer 1	FW2_a_b_rev	FW1_a_b_for/Link1_a_b_rev
	CDR2	CDR2_a_b	Megaprimer 2	–	Link1_a_b_for/Link2_a_rev
	CDR3	CDR3_a	–	–	Link2_a_for/FW4_a_b_rev
loop	CDR1	CDR1_a_b	Megaprimer 1	FW2_a_b_rev	FW1_a_b_for/Link1_a_b_rev
	CDR2	CDR2_a_b	Megaprimer 3	–	Link1_a_b_for/Link2_b_rev
	CDR3	CDR3_b	–	–	Link2_b_for/FW4_a_b_rev
convex	CDR1	CDR1_c	Megaprimer 4	FW2_c_rev	FW1_c_for/Link1_c_rev
	CDR2	CDR2_c	Megaprimer 5	–	Link1_c_for/Link2_c_rev
	CDR3	CDR3_c	–	–	Link2_c_for/FW4_c_rev

### Attachment of the flanking region for ribosome display

3.42, 3.6 and 3.77 µg of the ligated concave, loop and convex library, respectively, were used as template for PCR amplification using primer pairs Med_FX_for/Med_FX_rev for the concave and the loop library and Long_FX_for/Long_FX_for for the convex library. The amount of ligated libraries used as template for PCR amplification represented the bottleneck of the library construction with diversities corresponding to 9 × 10^12^ for each of the three sybody libraries. The resulting PCR product was cleaned up by PCR purification kit (Macherey-Nagel, Oensingen, Switzerland), digested by BspQI, and column purified again. The pRDV vector (Binz et al., 2004) was made compatible with FX cloning by amplifying the vector backbone using the primer pair RD_FX_pRDV_for/RD_FX_pRDV_rev1. The resulting PCR product was digested with BspQI, ligated with the *ccdB* kill cassette excised from the vector pBXCA3GH (Bukowska et al., 2015) (Addgene #47071) with the same enzyme and transformed into *E. coli* DB3.1. The DNA region between the stem loop and the ribosome binding site was shortened by amplifying the resulting vector with the 5’-phosphorylated primer pair pRDV_SL_for/pRDV_SL_rev and ligating the obtained PCR product. The resulting vector was called pRDV_FX5 and served as template to amplify the 5’ and 3’ nucleotide sequences required for in vitro translation of mRNA for ribosome display. This was performed by PCR amplification with primer pairs 5’_flank_for/5’_flank_rev and 3’_flank_for/tolAk_rev, respectively. The resulting PCR products were column purified, digested with BspQI and column purified again. The digested flanking regions (60 and 80 µg of the 5’ and 3’ flank, respectively) were ligated with 75 µg of the respective sybody library in a volume of 4 ml and using 80 µl T4 ligase (5 U/µl, ThermoFisher). After ligation, the DNA fragments were gel-purified and yielded 13.8, 22 and 20.5 µg ligation products corresponding to the flanked concave, loop and convex libraries, respectively. 10 µg of each ligation was amplified by PCR using primers 5’_flank_for and tolAk_2 in a 96 well plate and a total of 5 ml PCR reaction and the resulting PCR product was column purified, yielding 180, 188 and 192 for the concave, loop and convex libraries, respectively. 10 µg of each amplified library was in vitro transcribed using RiboMAX Large Scale RNA Production System (Promega) and yielded 1.5 mg of mRNA for each library.

### Purification and biotinylation of target proteins

The coding sequence of GFP was cloned using primer set GFP_FX_FV/GFP_FX_RV into FX vector pBXNH3. GFP was purified by Ni-NTA, followed by HRV 3C protease cleavage, rebinding on Ni-NTA and SEC in PBS. Chemical biotinylation of GFP was carried out in PBS using EZ-Link Sulfo-NHS-LC-Biotin (Thermo Fisher) and mass spectrometry analysis revealed that the reaction contained predominantly GFP having one biotin moiety attached. The coding sequence of MBP was cloned using primer sets MBP_FX_FW/MBP_FX_FW into FX vector pBXNH3CA (Bukowska et al., 2015), which results in a fusion protein consisting of an N-terminal His10-tag, a 3C protease cleavage site, MBP and a C-terminal Avi-tag. In order to produce Avi-tagged TM287/288, a GS-linker was first introduced into pBXNH3CA between the 3C cleavage site and the adjacent SapI site by amplifying the vector with the primer pair GS-Linker_FW (5’ phosphorylated) and GS-Linker_RV, each containing half of the GS-linker as overhang, and blunt-end ligation of the resulting PCR product. The resulting expression vector was called pBXNH3LCA. TM287/288 was cloned into pBXNH3LCA by FX cloning, which attaches a cleavable His10-tag to the N-terminus of TM287 separated by a GS-linker and an Avi-tag to the C-terminus of TM288. To produce Avi-tagged IrtAB, the coding sequence of *irtAB* was amplified from genomic DNA of *Mycobacterium thermoresistibile* DSM44167 using the primer set IrtAB_FX_FW/IrtAB_FX_RV and cloned into the expression vector pBXCA3GH (Bukowska et al., 2015). GFP, MBP-Avi, TM287/288-Avi and IrtAB-Avi were expressed in and purified from *E. coli* by Ni-NTA chromatography as described previously (Binz et al., 2004; Hohl et al., 2012). The enzymatic site-specific biotinylation of the Avi-tag was carried out at 4°C overnight using purified BirA in 20 mM imidazole pH 7.5, 200 mM NaCl, 10% glycerol, 10 mM magnesium acetate, 0.03% β-DDM in case of TM287/288 and IrtAB and a two-fold excess of biotin over the Avi-tag concentration. 3C protease was added as well to this reaction mixture to cleave off the His10-tag. Next day, the mixture was loaded onto Ni-NTA columns to remove the His-tag, BirA and the 3C protease. Biotinylated target proteins were eluted from the Ni-NTA column using TBS containing 30 mM imidazole and in case of TM287/288 and IrtAB 0.03% β-DDM. Finally, biotinylated target proteins were purified by SEC using a Superdex 200 Increase 10/300 GL (GE Healthcare) in TBS containing in case of TM287/288 and IrtAB 0.03% β-DDM.

Human ENT1(2–456) was expressed by transient transfection in HEK293 freestyle cells as wild-type, full length protein using a pCDNA3.1(+) base vector (Invitrogen) and synthesized, codon optimized genes cloned by Genewiz. The protein was designed as N-terminal fusion of His-GFP-3C-Avi with His being a 10-fold repeat of histidine, GFP is the enhanced green fluorescent protein, 3C is the 3C precision protease cleavage site (LEVLFQGP) and Avi the sequence corresponding to the Avi-tag (GLNDIFEAQKIEWHE). Biotinylation was performed in vivo during protein production by co-transfection of cells with two different pCDNA3.1(+) plasmids coding for the ENT1 construct and *E.coli* BirA ligase and by supplementing the medium with 50 µM biotin during fermentation. Cells were harvested in all cases 65 hr post transfection and were flash frozen at −80°C. For purification, cell pellets were thawed and resuspended in solubilization buffer containing 50 mM Tris-HCl pH 7.5, 300 mM NaCl and 1 Roche protein inhibitors complete tab per 50 ml of buffer in a 1 to 3 ratio (3 ml of buffer for 1 g of cells) under gentle agitation for 30 min until homogeneity. Subsequently, 1% (w/v) of lauryl maltose neopentyl glycol (LMNG) and 10 µM S-(4-Nitrobenzyl)−6-thioinosine (NBTI) were added to the suspension and incubated for 1 hr at 4°C. Supernatant was cleared by ultracentrifugation at 100’000xg using a Beckmann Ti45 rotor for 45 min and incubated overnight at 4°C with 15 ml of preconditioned TALON affinity resin under gentle stirring. Resin was collected by low speed centrifugation and washed with a total of 15 column volumes 20 mM Tris-HCl pH 7.5, 0.003% (w/v) LMNG, 20 mM imidazole, 300 mM NaCl and 10 µM NBTI several times. Using an empty pharmacia XK16 column, resin was collected and further washed with a buffer containing 20 mM Tris-HCl pH 7.5, 0.003% (w/v) LMNG, 20 mM imidazole, 300 mM NaCl, 10 µM NBTI and 15 µM dioleoyl-sn-glycero-3-phospho-L-serine (DOPS) lipid and washed a second time using the same buffer containing 40 mM imidazole. Protein was eluted at 300 mM imidazole and subjected to a desalt step on a 53 ml GE Hi Prep 26/10 desalting column using desalting buffer consisting of 20 mM Tris-HCl pH 7.5, 0.003% (w/v) LMNG, 300 mM NaCl, 10 µM NBTI and 15 µM DOPS. To remove the GFP-His tag and reduce protein glycosylation, 3C-Prescission protease was added at a concentration of 1 unit per 50 µg of protein together with 10 µg/ml of PNgaseF and 10 µg/ml Endo-alpha-N-acetylgalactosaminidase. The mixture was incubated overnight at 4°C and His-GFP tag removal was monitored by fluorescence-detection size-exclusion chromatography (FSEC) analysis. To completely remove GFP, protein was purified by an affinity purification step using a HiTrap TALON column collecting the flow-through. Subsequently, protein was concentrated using an Amicon filter unit at 30 kDa molecular weight cut-off to about 0.5–1 ml volume and further purified via size–exclusion chromatography using a Superdex 200 10/300 GL increase column equilibrated in the SEC buffer containing 20 mM Tris-HCl pH 7.5, 0.003% (w/v) LMNG, 300 mM NaCl, 10 µM NBTI and 15 µM DOPS. SEC fractions corresponding to the biotinylated ENT1 protein were concentrated on an Amicon filter unit with a cut-off of 30 kDa to a final concentration of 1.35 mg/ml and stored at −80 upon flash freezing in liquid nitrogen. Quality and biotin modification of the protein were analysed by LC-MS revealing close to complete biotinylation.

Human GlyT1 was cloned as a codon-optimized gene into a modified pOET1 base vector (Oxford Expression) by Genewiz and expressed either as a C-terminal 3C-GFP-His or C-terminal Avi-3C-GFP-His fusion in *Spodoptera frugiperda* (Sf9) cells. Cells were grown to 2 million cells/ml in Sf9III medium in 50 liter wave bags (Sartorius, 25 l maximal volume) and infected using 0.25–0.5% (v/v) of virus. 72 hr post infection at viabilities higher than 85%, cells were harvested by centrifugation at 3000 x g for 10 min and 4°C, washed in PBS and re-centrifuged for 20 min. Cells were filled in plastic bags and frozen by putting the bag in a −80°C freezer. Thawed biomass was further washed twice by resuspension and centrifugation for 20 min at 5000 x g in a buffer containing 50 mM Tris-HCl pH 7.5, 150 mM NaCl and 1 Roche Complete Tablet per 50 ml volume. Washed cells were resuspended for solubilization for 30 min at 4°C while stirring in a buffer containing 50 mM Tris-HCl pH 7.5, 150 mM NaCl, 30 mM imidazole pH 7.5, 1% (w/v) LMNG, 0.1% CHS, 15 µM DOPS and 100 µM Cmpd1, a more soluble analogon of the glycine transporter 1 reuptake inhibitor Bitopertin. The suspension was centrifuged at 100’000xg in a Ti45 rotor for 20 min at 4°C to collect supernatant. Protein was purified by batch purification using TALON affinity resin (GE Healthcare), incubated with resin for 16 hr under stirring at 300 rpm and then centrifuged at 500xg for 2 min in a 50 ml Falcon tube. Four wash steps with a buffer containing 50 mM Tris-HCl, 150 mM NaCl, 30 mM imidazole pH 7.5, 0.05% (w/v) LMNG, 0.005% (w/v) CHS, 15 µM DOPS, 50 µM Cmpd1 and 1 Roche Complete Tablet per 50 ml volume were followed by loading the resin into a XK26 column (GE Healthcare), washed again with four column volumes to finally elute the protein with the same buffer that contained 300 mM imidazole. The Avi-3C-GFP-His but not the 3C-GFP-His protein was treated with HRV-3C protease (Novagen) and in-house produced PNGase F (*F. meningosepticum*) to cleave the GFP-His tag and trim existing glycosylations. Subsequently, the Avi-tagged GlyT1 was desalted into a buffer optimal for enzymatic biotinylation consisting of 20 mM bicine pH 8.3, 150 mM potassium-glutamate pH 7.5, 0.05% (w/v) LMNG, 0.005% CHS, 15 µM DOPS and 50 µM Cmpd1 to remove imidazole and subjected to another round of TALON affinity purification to remove the cleaved GFP-His tag in the same buffer. The flow through containing the GlyT1 protein was concentrated to 1.1 mg/ml concentration with an Amicon Ultra four filter unit (Millipore) with a molecular weight limit of 30 kDa for complete biotinylation using the BirA-500 biotinylation kit (Avidity) according to the protocol. Biotinylation was monitored by liquid-chromatography-coupled mass spectroscopy. Both the Avi fusion as well as the 3C-GFP-His fusion protein were further purified via size-exclusion chromatography using a Superdex 200 10/300 GL increase column and a buffer containing 50 mM Tris-HCl pH 7.5, 150 mM NaCl, 0.05% (w/v) LMNG, 0.005% (w/v) CHS, 15 µM DOPS and 50 µM Cmpd1.

### Sybody selections against MBP

To display sybody libraries on ribosomes, 10 µl of the PURE*frexSS* translation mix was prepared (GeneFrontier Corporation, Kashiwa, Japan). The kit components were mixed to a total volume of 9 µl and incubated at 37°C for 5 min. 1 µl of 2 µM library RNA was added to the translation mix and incubated at 37°C for 30 min. The ribosomal complexes were diluted in 100 µl ice cold WTB buffer (50 mM Tris-acetate pH7.5, 150 mM NaCl, 50 mM magnesium acetate) supplemented with 0.05% Tween 20, 0.5% BSA and 5 mg/ml Heparin. 10 µl Dynabeads MyOne Streptavidin T1 (Life Technologies) were washed 3 times with 500 µl WTB and blocked with 500 µl WTB 0.5% BSA for 1 hr followed by 3 washes 500 µl of WTB-T-BSA (0.05% Tween 20, 0.5% BSA). The magnetic beads were coated in 100 µl WTB-T-BSA containing 50 nM biotinylated MBP for 1 hr followed by 3 washes of 500 µl WTB-T-BSA. The ribosomal complexes were incubated with the beads for 20 min followed by 3 washes of 500 µl WTB-T. During the last wash step, the beads were placed in a fresh tube. The RNA was eluted by resuspending the beads in 100 µl TBS supplemented with 50 mM EDTA pH 8.0 and 100 µg/ml yeast RNA and incubated for 10 min at room temperature. The eluted RNA was purified using the RNeasy micro kit (Qiagen) and eluted in 14 µl RNase-free water. Reverse transcription was performed by mixing 14 µl of the eluted RNA with 2 µl of RT_Primer at 100 µM and 4 µl of 10 mM dNTPs. The mixture was heated to 65°C for 5 min, and then cooled on ice. Using this mixture, a 40 µl RT reaction was assembled according to the manual (Affinity Script, Agilent) and incubated 1 hr at 37°C, followed by 5 min at 95°C. The cDNA was purified using the PCR purification kit (Macherey Nagel) and eluted in 30 µl elution buffer. 25 µl of the purified cDNA was amplified by PCR using Q5 High-Fidelity DNA Polymerase (NEB) and primers Medium_ORF_for and Medium_ORF_rev for the concave and loop library, and Long_ORF_for and Long_ORF_rev for the convex library, respectively. The PCR product was purified via gel and used as template in an assembly PCR to add the flanking regions for in vitro transcription using megaprimers. Megaprimers to flank the concave and loop sybodies were obtained by amplifying pRDV_FX5 containing the non-randomized loop sybody using primer pairs 5'_flank_for/Medium_ORF_5’_rev and Medium_ORF_3’_for/tolAk_rev. Megaprimers to flank the convex sybodies were obtained by amplifying pRDV_FX5 containing the non-randomized convex sybody using primer pairs 5'_flank_for/Long_ORF_5’_rev and Long_ORF_3’_for/tolAk_rev. Flanking was performed by assembly PCR using 200 ng sybody pool obtained from RT-PCR, 120 ng of 5’-flank, 360 ng of 3’-flank and 5 µM of outer primers 5'_flank_for and tolAk_2 in a volume of 100 µl. The resulting PCR product was separated on gel, purified and used as input material for 10 µl reaction of the RiboMAX Large Scale RNA Production System (Promega). The resulting RNA was purified using the RNeasy kit (Qiagen) and used as input RNA of the next round. The second round was performed according to the first round. In the third round, the PCR product of the amplified cDNA was amplified using primers Med_FX_for/Med_FX_rev (concave and loop library) or Long_FX_for/Long_FX_for (convex library) to add FX overhangs to the DNA and subsequently cloned into the pSb_init vector by FX cloning for expression and ELISA.

### Sybody selections against membrane proteins

For clarity of discussion, we describe here only the final form of the selection protocol and do not specify how we gradually evolved the selection method as outlined in Figure 1—figure supplement 6. One round of ribosome display was performed as in the MBP section with the following exceptions. Tween 20 was replaced by 0.1% β-DDM for selections against TM287/288(E517A), 0.1% β-DDM and 0.005% CHS against ENT1, or by 0.05% LMNG and 0.005% CHS against GlyT1. Selections against TM287/288(E517A), ENT1 and GlyT1 were carried out in the presence of 1 mM ATP, 10 µM NBTI and 50 nM Cmpd1, respectively. Solution panning was performed by incubating ribosomal complexes and 50 nM biotinylated target protein for 30 min prior to the pulldown via streptavidin coated magnetic beads (Dynabeads MyOne Streptavidin T1). The cDNA was amplified using GoTaq G2 DNA Polymerase (Promega) and primers Med_FX_for/Med_FX_rev for concave/loop sybodies or Long_FX_for/Long_FX_rv for the convex sybodies. The resulting PCR product was cloned into the phagemid vector pDX_init. To this end, amplified sybody pools and pDX_init were digested with BspQI, gel-purified and 500 ng sybody insert and 1 µg pDX_init backbone were ligated in 50 µl using 5 units of T4 DNA Ligase (Thermo Scientific, Reinach, Switzerland). The ligation reaction was mixed with 350 µl electrocompetent *E.coli* SS320 on ice. After electroporation (Bio Rad Gene Pulser, 2.5 kV, 200 Ω, 25pF), the cells were immediately resuspended in 25 ml SOC and shaken at 37°C for 30 min for recovery. Subsequently, the cells were diluted in 250 ml 2YT, 2% glucose, 100 µg/ml ampicillin and grown overnight shaking at 37°C. For phage production, this phagemid-containing *E.coli* SS320 overnight culture was inoculated 1:50 in 50 ml 2YT, 2% glucose, 100 µg/ml ampicillin and grown to OD_600_ of 0.5. 10 ml of this culture was superinfected with 3 × 10^11^ plaque forming units M13KO7 Helper Phage at 37°C without shaking for 30 min. Cells were collected by centrifugation and resuspended in 50 ml 2YT, 100 µg/ml ampicillin, 25 µg/ml kanamycin and incubated at 37°C shaking overnight for phage production. Next day, cells were pelleted by centrifugation and 40 ml of the culture supernatant were mixed with 10 ml 20% PEG6000 (v/v), 2.5 M NaCl and incubated on ice for 30 min to precipitate the phages, which were subsequently pelleted by centrifugation. The pellet was resuspended in phosphate-buffered saline (PBS) and cleared twice by centrifugation in a tabletop centrifuge at full speed. Phage concentration was determined by UV-Vis spectroscopy. The first round of phage display was performed in a Maxi-Sorp plate (Nunc) using 48 wells per target. The plate was coated overnight with 100 µl per well of 60 nM neutravidin in TBS. Next day, the plate was washed three times with TBS and blocked with TBS containing 0.5% BSA for 1 hr. The phages were diluted to 10^12^ phages per ml in ice cold TBS-D-BSA (containing 0.1% β-DDM for TM287/288(E517A), 0.1% β-DDM and 0.005% CHS and ENT1 or 0.05% LMNG and 0.005% CHS for GlyT1, and 0.5% BSA for all targets). 4.8 ml of this phage stock was incubated in solution for 20 min with 50 nM biotinylated target protein (in the presence of 1 mM ATP +2 mM MgCl_2_ for TM287/288(E517A), 10 µM NBTI for ENT1 or 50 nM Cmpd1 for GlyT1). The plate was prepared by washing three times with ice cold TBS-D-BSA and 100 µl of the phage/target mix was added per well (4.8 ml in total added to 48 wells) and incubated for 10 min at 4°C. The plate was subsequently washed 3 times with 250 µl/well ice cold TBS-D (devoid of ligands and BSA). Phages were eluted by adding 100 µl TBS with 0.25 mg/ml trypsin per well and incubation at room temperature for 30 min (total elution volume of 4.8 ml). Trypsin was inhibited by adding 0.125 mg/ml 4-(2-Aminoethyl)benzenesulfonyl fluoride (AEBSF) to the elution. For infection, a culture of *E.coli* SS320 was grown in 2YT to an OD_600_ of 0.5. 4.8 ml of eluted phages was added to 50 ml of the culture and incubated at 37°C without shaking for 30 min. The cells were then diluted 1:10 in 500 ml 2YT, 2% glucose, 100 µg/ml ampicillin and grown overnight shaking at 37°C resulting in a preculture for phage production for the next round. The second round of phage display was performed according to the first one except that 10 μl magnetic beads (Dynabeads MyOne Streptavidin C1) were used to pull down target-phage complexes. The total working volume was reduced from 4.8 ml to 100 μl and beads were washed three times with 500 μl TBS-D. Phages were eluted in 100 μl and used to infect 1.4 ml *E.coli* SS320. After infection, cells were diluted 1:10 in 15 ml 2YT, 2% glucose, 100 µg/ml ampicillin and grown overnight shaking at 37°C. The overnight culture was used to purify the phagemids (MiniPrep, Qiagen) containing the selected sybodies. Sybody sequences were subcloned into pSb_init using FX cloning and transformed into *E. coli MC1061* for ELISA analysis and protein purification.

### Sybody identification by ELISA

Single sybody clones were picked and expressed in 1 ml terrific broth containing 25 µg/ml chloramphenicol in a 2 ml 96 deep well plate. After expression, the cells were pelleted by centrifugation and resuspended in 50 µl B-PER II for lysis. The lysate was diluted with 950 µl TBS and centrifuged to pellet cell debris. ELISAs were carried out in Maxi-Sorp plates (Nunc) coated overnight with 100 µl/well of 5 µg/ml Protein A in TBS. The plate was washed 3 times with 250 µl TBS and blocked with 250 µl TBS-BSA. All washing steps were performed using 3 times 250 µl TBS containing detergent (0.05% Tween-20 for MBP; 0.03% β-DDM for TM287/288; 0.03% β-DDM/0.003% CHS for ENT1; 0.005% LMNG/0,0005% CHS for GlyT1) between all incubation steps, which were carried out in 100 µl TBS-D-BSA for 20 min. These steps were anti-myc antibody 1:2000 (Sigma Aldrich, Buchs, Switzerland, M4439) followed by five fold diluted sybody lysate, then 50 nM of the biotinylated target protein or biotinylated control protein and finally streptavidin-HRP 1:5000 (Sigma Aldrich, S2438) (Figure 1). The ELISA was developed by adding 100 µl of 0.1 mg/ml TMB (Sigma Aldrich 860336) in 50 mM Na_2_HPO_4_, 25 mM citric acid and 0.006% H_2_O_2_. ELISA signals were measured at an absorbance of 650 nm.

### Monitoring of binder enrichment by qPCR

With the term ‘enrichment’, we refer to the experimentally determined fold excess of polynucleotides eluted from a selection round against a target of choice versus an analogous selection round against another immobilized protein, which was not used for selections in preceding rounds. In order to determine enrichments, qPCR was performed in a 10 µl reaction containing SYBR select Master Mix (Thermo Fischer Scientific), 300 nM of each primer, 5% DMSO and 2 µl cDNA (ribosome display) or phages (phage display) diluted 10 fold in H_2_O. Standard curves for each primer pair were determined using a dilution series of the phagemid pDX_init or a PCR product corresponding to the sequence of the cDNA obtained after ribosome display. Their initial concentration was determined by UV-Vis spectroscopy. PCR efficiencies for all primer pairs were between 95 and 98%. Cycling conditions were: 2 min 95°C initially for polymerase activation, followed by 10 s 95°C and 30 s 63°C for 45 cycles. The runs were performed in a 7500 fast qPCR machine (Thermo Fischer Scientific). The following primer pairs were used: qPCR_RD_5’_for in combination with qPCR_ RD_S and M_5’_rev for the concave and loop library or qPCR_ RD_L_5’_rev for the convex library to determine the amount of full length cDNA, qPCR_ RD_tolA_3’_for/qPCR_ RD_tolA_3’_rev to determine the total amount of cDNA, qPCR_PD_pDX_for/qPCR_PD_pDX_rev to determine the amount of phages and qPCR_3K1K_for/qPCR_3K1K_rev to determine the amount of 3K1K cDNA. The qPCR reactions were performed as technical triplicates.

### Protein stability measurements using thermofluor

Thermofluor was performed in a 25 µl reaction of PBS containing 100x SYPRO Orange (Life Technologies) and 0.5 mg/ml sybody. The reaction was heated with a 1% ramp from 25 to 99°C in a 7500 fast qPCR machine (Thermo Fischer Scientific) while the fluorescence intensity was measured through a ROX filter. The raw data were extracted and fitted as described previously (Ericsson et al., 2006). Two technical replicates were performed for each protein and one representative dataset was fitted.

### Surface plasmon resonance

Binding affinities were determined using surface plasmon resonance (SPR). MBP binders were analyzed using a Biacore X100 machine (GE healthcare). 570 response units (RU) of biotinylated MBP were immobilized on a streptavidin coated SPR chip (Sensor Chip SA). Sybodies Sb_MBP#1–3 were purified in TBS, 0.05% Tween-20 as described above. SPR measurements were carried out as technical triplicates for each sybody concentration in the same buffer. Affinities of Sb_MBP#1 were in addition determined in the same buffer supplemented with increasing maltose (4-*O*-α-D-Glucopyranosyl-D-glucose) concentrations (0, 5, 10, 25, 50 and 100 µM). In these analyses, 860 response units (RU) of biotinylated MBP were immobilized, and traces for each sybody concentration was measured once. SPR data were fitted with a 1:1 interaction model using the Biacore X100 evaluation software and further analyzed by plotting sybody affinity ratios determined in the presence (*K*_D_’) and absence (*K*_D_) of maltose against the maltose concentration and fitting the data with the following equation:

$$y=\frac{\frac{x}{B}+1}{\frac{\alpha x}{B}+1}$$

B corresponds to the binding affinity of maltose (*K*_D,maltose_) and α corresponds to the allosteric constant.

Affinities of sybodies directed against TM287/288 were measured using a ProteOn XPR36 Protein Interaction Array System (Biorad). Biotinylated TM287/288 and TM287/288(E517A) mutant were immobilized on a ProteOn NLC Sensor Chip at a density of 1500 RU. Sybodies expressed in pSb_init were SEC-purified in TBS, and SPR analysis was carried out in the same buffer containing 0.015% β-DDM and either 1 mM MgCl_2_ or 1 mM MgCl_2_ +0.5 mM ATP, to measure binding affinities in the presence or absence of ATP. Traces for each sybody concentration were recorded once and the data were fitted with a 1:1 interaction model using the BioRad Proteon Analysis Software.

All ENT1 and GlyT1 SPR experiments were performed on a Biacore T200 (GE Healthcare, Uppsala, Sweden) instrument at 18°C in running buffers containing either 20 mM HEPES pH 7.5, 150 mM NaCl, 0.001% (w/v) LMNG, 1.25 µM DOPS (ENT1) or 20 mM Citrate pH 6.4, 150 mM NaCl, 0.004% (w/v) LMNG (GlyT1) at 50 µl/min flow rate. Running buffers were freshly prepared, filtered with ExpressPlus steritop filters with 0.22 µm cut off (Millipore, Billerica, MA, USA) and degassed prior the SPR analysis. Biotinylated ENT1 or GlyT1 were captured on streptavidin pre-coated SA sensors (GE Healthcare BR-1000–32). First, streptavidin sensors were conditioned with 3 consecutive 1 min injections of high salt solution in sodium hydroxide (50 mM NaOH, 1 M NaCl). Next, biotinylated protein samples were applied to a streptavidin sensor surface for protein immobilization levels of about 1000 RU and 300 RU for ENT1 and GlyT1, respectively. Finally, free biotin solution (10 µM in running buffer) was injected once (1 × 1 min) over the sensor surface to block remaining streptavidin binding sites. Dose-response experiments were performed at sybody concentrations up to 475 nM (ENT1) and up to 2 µM (GlyT1). All monitored resonance signals were single referenced, i. e. signals monitored on the binding active channel were subtracted with signals from a reference channel. SPR measurements on ENT1 and GlyT1 were carried out using two independently coated SPR chips and were highly reproducible. One of the two datasets is shown. Data fitting was performed with a 1:1 interaction model using the Biacore T200 Evaluation (v2.0) software.

### Biolayer interferometry

The Octet RED96 System (FortéBio, Pall Inc.) uses disposable sensors with an optical coating layer immobilized with streptavidin at the tip of the sensor. Sensors were decorated with biotinylated MBP to reach a stable baseline, arbitrarily set to 0 nm (Figure 2—figure supplement 4). Sensors were dipped in a well containing 500 nM Sb_MBP#1 which leads to the formation of the Sybody-MBP complex. Sensors containing the complex were sequentially dipped in a row of wells containing 500 nM Sb_MBP#1 and increasing concentrations of maltose (0.1, 1, 10, 100, 1000 µM). The reversibility of the competition was shown by decreasing maltose concentrations (1000, 100, 10, 1, 0.1, 0 µM) again in the presence of 500 nM Sybody Sb_MBP#1. The experiments were done in technical duplicates.

### Thermal shift scintillation proximity assay (SPA-TS) using tritiated small molecule inhibitors

Ligand binding assays were performed in a buffer containing either 50 mM Tris-HCl pH 7.5, 150 nM NaCl and 0.004% (w/v) LMNG or 20 mM Citrate pH 6.4, 150 mM NaCl and 0.004% (w/v) LMNG, respectively. For each analysis, 140 µl protein solution (7 nM for ENT1 and 10 nM for GlyT1) was added to each of 12 wells of a 96-well Eppendorf PCR plate at 4°C and incubated subsequently for 10 min with a temperature gradient from 30–60°C (ENT1) or 23–53°C (GlyT1) across twelve wells in a Techne Prime Elite thermocycler. Subsequently, the plate was centrifuged at 2250 x g for 3 min at 4°C and 135 µl of protein solution transferred to a 96-well Optiplate (Perkin Elmer) preloaded with 15 µl Copper SPA beads (20 mg/ml) per well (PerkinElmer) to obtain a final bead concentration of 0.3 mg/well. After 15 min of incubation at 4°C and 1000 rpm on a BioShake iQ, a final concentration of 6 nM tritiated [^3^H]-NBTI (Perkin-Elmer) or [^3^H]-Org24598 compound (1.2mCi/ml specific activity, 50 µl of a 24 nM stock solution) was added and incubated for 45 min at 4°C and 1000 rpm on a Bioshake iQ. Scintillation analysis was performed using a TopCount Microplate Scintillation Counter. Ent1 measurements were performed with three technical replicates for each temperature, whereas single measurement points for each temperature were determined for GlyT1. Apparent melting temperatures (*T*_m_s) were determined in GraphPad Prism 6.07 using a non-linear fit to a Boltzmann sigmoidal function.

### ATPase inhibition of TM287/288

ATPase activity was measured as described previously (Hohl et al., 2014) in TBS containing 0.03% β-DDM and 10 mM MgSO_4_ at increasing concentrations of sybody Sb_TM#26. ATP concentration was 50 µM, TM287/288 concentration was 25 nM, assay temperature was 25°C and incubation time was 20 min. Each data point was determined as technical triplicate. The data were fitted to a hyperbolic decay curve with the following function (SigmaPlot):

$$y=y_{0}+\;\frac{a\;IC_{50}}{IC_{50}+x}$$

in which *y* corresponds to the ATPase activity at the respective sybody concentration divided by the ATPase activity in the absence of inhibitor normalized to 100%, *IC_50_* corresponds to the sybody concentration for half-maximal inhibition, *y_0_* corresponds to the residual activity at infinite sybody concentration, *a* corresponds to the maximal degree of inhibition, and *x* corresponds to the sybody concentration.

### Data availability

The sybody-MBP structures have been deposited at the Protein Data Bank under the accession codes 5M13, 5M14 and 5M15. Vectors have been deposited at Addgene. All other data generated or analyzed during this study are included in this published article.

## Additional files

Transparent reporting form

### Data availability

Diffraction data have been deposited in PDB under the accession codes 5M13, 5M14 and 5M15. Vectors have been deposited at Addgene under accession IDs #110098 - #110104

The following previously published datasets were used:

Iwan ZimmermannPascal EgloffMarkus A Seeger2017Synthetic nanobody in complex with MBPhttps://www.rcsb.org/structure/5M13Publicly available at the RCSB Protein Data Bank (accession no. 5M13)

Iwan ZimmermannPascal EgloffMarkus A Seeger2017Synthetic nanobody in complex with MBPhttps://www.rcsb.org/structure/5M14Publicly available at the RCSB Protein Data Bank (accession no. 5M14)

Iwan ZimmermannPascal EgloffMarkus A Seeger2017Synthetic nanobody in complex with MBPhttps://www.rcsb.org/structure/5M15Publicly available at the RCSB Protein Data Bank (accession no. 5M15)
