# Supplementary material for: Synthetic single domain antibodies for the conformational trapping of membrane proteins
Source: eLife. 2018 May 24;7:e34317. doi: 10.7554/eLife.34317 (PMC5967865; doi:10.7554/eLife.34317)
Supplement: Transparent reporting form [file elife-34317-transrepform.pdf]

1st Floor
24 Hills Road
Cambridge CB2 1JP, UK

P 01223 855340
W elifesciences.org
T @elife

eLife's transparent reporting form

We encourage authors to provide detailed information *within their submission* to facilitate the interpretation and replication of experiments. Authors can upload supporting documentation to indicate the use of appropriate reporting guidelines for health-related research (see EQUATOR Network), life science research (see the BioSharing Information Resource), or the ARRIVE guidelines for reporting work involving animal research. Where applicable, authors should refer to any relevant reporting standards documents in this form.

If you have any questions, please consult our Journal Policies and/or contact us: editorial@elifesciences.org.

Sample-size estimation

- You should state whether an appropriate sample size was computed when the study was being designed
- You should state the statistical method of sample size computation and any required assumptions
- If no explicit power analysis was used, you should describe how you decided what sample (replicate) size (number) to use

Please outline where this information can be found within the submission (e.g., sections or figure legends), or explain why this information doesn't apply to your submission:

Sample size estimation was not relevant for this study, as it does not report on a statistical evaluation of effects between two or more groups.

Replicates

- You should report how often each experiment was performed
- You should include a definition of biological versus technical replication
- The data obtained should be provided and sufficient information should be provided to indicate the number of independent biological and/or technical replicates
- If you encountered any outliers, you should describe how these were handled
- Criteria for exclusion/inclusion of data should be clearly stated
- High-throughput sequence data should be uploaded before submission, with a private link for reviewers provided (these are available from both GEO and ArrayExpress)

Please outline where this information can be found within the submission (e.g., sections or figure legends), or explain why this information doesn't apply to your submission:

eLife Sciences Publications, Ltd is a limited liability non-profit non-stock corporation incorporated in the State of Delaware, USA, with company number 5030732, and is registered in the UK with company number FC030576 and branch number BR015634 at the address 1st Floor, 24 Hills Road, Cambridge CB2 1JP | August 2014

All relevant information can be found in the Material and methods section as well as in the Figure legends.

Binder selections against target proteins were carried out with two technical replicates, giving very similar degrees of binder enrichment as judged by pPCR.

ELISA hits were replicated in a cross-specificity ELISA where the sybodies were tested against an array of target and dummy proteins.

qPCR runs were performed as technical triplicates and were highly reproducible. The obtained CT values were averages of these triplicates.

Thermal unfolding using Sypro Orange was performed by two technical replicates, which were highly reproducible. For fitting, representative datasets were used (see legend Figure 1–Figure Supplement 3).

SPR measurements on the MBP sybodies contained three technical replicates for each sybody concentration (see legend Figure 2–Figure Supplement 4). The TM287/288 sybodies were measured by SPR (Biorad ProteOn) with one replicate for each sybody concentration. SPR measurements for ENT1 and GlyT1 sybodies were carried out twice on different SPR chips, but using the same biological material and were highly reproducible. Representative data are shown in this case.

Each SPA-TS measurement (i.e. in presence or absence of syobdy) is based on 12 data points, which were in case of ENT1 determined as technical triplicates (error bars in Figure 5C are standard deviations) or in case of GlyT1 as single measurements.

ATPase activity measurements were carried out with three technical replicates for each data point (i.e. each sybody concentration) and these data were used to calculate the average and standard deviations (see legend Figure 3).

eLife Sciences Publications, Ltd is a limited liability non-profit non-stock corporation incorporated in the State of Delaware, USA, with company number 5030732, and is registered in the UK with company number FC030576 and branch number BR015634 at the address 1st Floor, 24 Hills Road, Cambridge CB2 1JP | August 2014

Statistical reporting

- Statistical analysis methods should be described and justified
- Raw data should be presented in figures whenever informative to do so (typically when N per group is less than 10)
- For each experiment, you should identify the statistical tests used, exact values of N, definitions of center, methods of multiple test correction, and dispersion and precision measures (e.g., mean, median, SD, SEM, confidence intervals; and, for the major substantive results, a measure of effect size (e.g., Pearson's r, Cohen's d)
- Report exact p-values wherever possible alongside the summary statistics and 95% confidence intervals. These should be reported for all key questions and not only when the p-value is less than 0.05.

Please outline where this information can be found within the submission (e.g., sections or figure legends), or explain why this information doesn't apply to your submission:

All error bars are clearly defined as standard deviations. This information is given in the Figure legend (where appropriate) as well as in the Material and Methods section.

(For large datasets, or papers with a very large number of statistical tests, you may upload a single table file with tests, Ns, etc., with reference to sections in the manuscript.)

Group allocation

- Indicate how samples were allocated into experimental groups (in the case of clinical studies, please specify allocation to treatment method); if randomization was used, please also state if restricted randomization was applied
- Indicate if masking was used during group allocation, data collection and/or data analysis

Please outline where this information can be found within the submission (e.g., sections or figure legends), or explain why this information doesn't apply to your submission:

The experiments described in this paper did not require group allocation.

Additional data files ("source data")

- We encourage you to upload relevant additional data files, such as numerical data that are represented as a graph in a figure, or as a summary table
- Where provided, these should be in the most useful format, and they can be uploaded as "Source data" files linked to a main figure or table
- Include model definition files including the full list of parameters used
- Include code used for data analysis (e.g., R, MatLab)
- Avoid stating that data files are "available upon request"

Please indicate the figures or tables for which source data files have been provided:

The structures of the sybody-MBP complexes were deposited on the protein database (PDB).
