# Supplementary material for: Synthetic single domain antibodies for the conformational trapping of membrane proteins
Source: eLife. 2018 May 24;7:e34317. doi: 10.7554/eLife.34317 (PMC5967865; doi:10.7554/eLife.34317)
Supplement: Peer review history

# Decision letter

**DOI:** 10.7554/eLife.34317.039

**Affiliations:** NINDSUnited States

In the interests of transparency, eLife includes the editorial decision letter and accompanying author responses. A lightly edited version of the letter sent to the authors after peer review is shown, indicating the most substantive concerns; minor comments are not usually included.

[Editors’ note: this article was originally rejected after discussions between the reviewers, but the authors were invited to resubmit after an appeal against the decision.]

Thank you for submitting your work entitled "Synthetic single domain antibodies for the conformational trapping of membrane proteins" for consideration by *eLife*. Your article has been reviewed by three peer reviewers, and the evaluation has been overseen by a Reviewing Editor and a Senior Editor. The following individuals involved in review of your submission have agreed to reveal their identity: Simon Newstead (Reviewer #1); Rachelle Gaudet (Reviewer #2).

Our decision has been reached after consultation between the reviewers. Based on these discussions and the individual reviews below, we regret to inform you that your work will not be considered further for publication in *eLife*.

The reviewers found the new library to be of potential interest to the membrane protein community but noted that claims regarding the factors contributing to the reported success of the technique were unsubstantiated, and the claimed technical advance of the platform over previously published synthetic nanobody libraries is not supported with direct evidence. We therefore recommend that the article be sent to a more specialized journal.

Reviewer #1:

I would like to declare that we are currently collaborating with Prof. Seeger's group to apply this technology to several of our current targets.

That being said, I find the present manuscript to contain highly relevant and important information in the design and deployment of an important and very useful tool for membrane protein structural biologists and biochemists. I am in favor of publication with minimal additional work.

The work is clearly explained, and the results are compelling in my opinion. The strategy followed is very similar to that used for the generation of monobody libraries by Prof. Koide and antibody libraries by Prof. Kossiakoff but applied to the single chain nanobodies that have proved so useful in several structural studies on membrane proteins. In particular the authors use ribosome display instead of phage display to generate a larger library and explain well how they used a commercial in vitro translation kit to leverage this technology.

It is also notable that the authors are willing to make their library available to other academic labs, which will significantly improve uptake of this technology within the structural biology community.

There are many reasons why groups may wish to establish this technology, whether as crystallisation chaperones or as conformational specific biochemical clamps for biophysical studies. I think the present work is well written and supported by good examples, including the crystal structures of these scaffolds bound to MBP.

However, in several places in the text the authors refer to the successful use of the technology in determining the outward facing state of the ABC transporter, MsbA. I am not sure whether *eLife* policy will allow (unpublished) results to support current work? I guess the major soft spot in the study is the lack of a true membrane protein co-crystal structure (in the absence of the MsbA structure), but the study has been submitted under the 'Tools' heading, so I am not sure this is necessary. The technology works and is easy to use.

Reviewer #2:

This manuscript by Zimmermann and colleagues describes a set of three "sybody" libraries based on recently published nanobodies that were successfully used to determine structures of proteins in specific conformations. The authors describe important features and parameters that they optimized to obtain libraries that produce useful binders. The libraries are also validated with multiple targets, ranging from the simple/easy target (MBP) to much more challenging ones, including two eukaryotic SLC transporters. Overall, the libraries performed very well, and the results with the ABC transporter are particularly impressive. The advantages and potential pitfalls, and the reasoning for specific choices are all well described and provide a valuable resource for others considering building or using a similar library.

I could not find the nucleotide mixtures used for the three mixes at the randomized codons anywhere in the manuscript (the legend to Figure 1—figure supplement 2 states "Details are provided in Supplementary results." But no Supplementary results were included, and this was the only reference to such Supplementary results. These mixtures need to be described in the manuscript as they are an important feature of the libraries.

The Materials and methods section describing the construction of the vectors and libraries are rather challenging to follow. The authors should consider reorganizing or perhaps reformatting so that the stepwise process is made clearer.

At the end of the Discussion section, the authors state that "The libraries and protocols will be made fully available to academic labs in order to facilitate the spread and further development of the technology." This statement would be much more effective if the relevant scaffold plasmids and other vectors used were available through a service like Addgene and the database numbers were directly included in this manuscript.

Reviewer #3:

The work is a logical, incremental improvement in binder generation by integrating several lines of prior studies over the last two decades. Although the developed libraries seem effective and the manuscript provides detailed descriptions of the study, this work does not critically evaluate the technology or provides mechanistic insights into the protein systems studied. The manuscript overstates innovation and impact of this study and makes many unsupported statements.

The authors attribute their successes to three factors, large library size, selection using both ribosome display and phage display, and in vitro selection at low temperature and in the presence of inhibitors. However, the authors provide no supporting evidence for these claims. Similarly, the authors imply superiority of their platform over previously published synthetic nanobody libraries with no direct evidence (Discussion section).

The description of the challenges in binder generation (Introduction) seem inaccurate. Many synthetic antibodies to membrane proteins seem to have been generated by Kossiakoff and colleagues, straight from a synthetic antibody library without "extensive binder screening and purification efforts".

Subsection “The three binding modes of sybodies”. The designs of the three libraries with different paratope topography closely follow the library designs of non-antibody scaffolds (DARPins, Anticalins, Monobodies) over the last decade (PMID 19416843, 22198408, 24513107). This work is particularly similar in concept to PMID 22198408. These precedents should be properly described in this section.

Establishment of the sybody framework and randomization strategy closely follows the concept developed for synthetic libraries on a single antibody framework where only exposed CDR positions are diversified (PMID 15066433 in 2004 and subsequent papers). Thus, the authors' apparent claim of novelty in the Discussion section is unsupported. Similarly, the use of biased and restricted amino acid diversity with emphasis on Tyr and Ser has been well established (PMID 15306681, 15854651, 16413576, 17420456).

The authors should compare properties of the MBP-binding sybodies with those of many existing binders to MBP (DARPin, monobodies and Fabs). The authors do not mention that high-affinity monobodies have been generated against MBP from extremely simple and small libraries with as few as 10^6^ members (PMID 17420456, 18602117). These monobodies and the reported sybodies appear to share similar binding modes. Conformation-specific antibodies have been generated for MBP directly from a synthetic phage-display library (PMID 21378967). Therefore, the success in generating MBP-binding sybodies does not offer much support for the effectiveness of the library designs or selection methodology. Why do the sybodies have strong preference to the cleft, although the libraries should present distinct topographies. In contrast, the DARPin and Fabs appear to prefer binding outside the cleft (PMID 15097997; PDB 5BJZ, 5BK1, 5BK2).

It is unclear why the authors did not use nonhydrolyzable ATP analogs in the selection for TM287/288 or for stabilizing its outward-facing state. The authors used a threshold of just threefold in affinity (corresponding to ~0.7 kcal/mol) for designating conformation-specific binders. Binders with such low specificity are not likely to be useful in biophysical and structural applications. Using a more realistic threshold of tenfold, only five clones would be considered specific. The conclusion for this section (subsection “Conformational trapping of a bacterial ABC transporter”) is an overstatement.

Much of the Discussion section repeats descriptions of results and thus adds little to the manuscript. They should be omitted or drastically shortened.

The authors should include the full sequences of sybodies to GlyT1 and ENT1. For GlyT1, the authors should describe whether clones from a particular library design preferentially bind to one or the other epitope (Figure 6D).

[Editors’ note: what now follows is the decision letter after the authors submitted for further consideration.]

Thank you for resubmitting your work entitled "Synthetic single domain antibodies for the conformational trapping of membrane proteins" for further consideration at *eLife*. Your revised article has been favorably evaluated by Arup Chakraborty (Senior Editor) and a Reviewing Editor.

The manuscript has been significantly improved but there are some remaining issues that need to be addressed before acceptance, as outlined below:

- In the new Figure 1—figure supplement 6, there is a discrepancy, in that the panel C describes analysis of lrtAB, whereas panels A, B, and D describe results for TM287/288, suggesting that the results are not comparable. Ideally TM287/288 would be tested using the same protocol, although, since these data were motivational rather than demonstrative, this is not in our view, essential. Nevertheless, to gloss over the discrepancy raises concerns. The authors could instead mention, e.g., that the results came from a separate study. And/or they could also remove the arrows from the figure, which imply that they came from a single experimental design process.

- Please (briefly) expand on the explanation of solution panning and the alternate surface selection strategy within the main flow of the manuscript (subsection “Conformational trapping of a bacterial ABC transporter”).

# Reply: Author response

**DOI:** 10.7554/eLife.34317.040

[Editors’ note: the author responses to the first round of peer review follow.]

Reviewer #1:

[…] However, in several places in the text the authors refer to the successful use of the technology in determining the outward facing state of the ABC transporter, MsbA. I am not sure whether eLife policy will allow (unpublished) results to support current work? I guess the major soft spot in the study is the lack of a true membrane protein co-crystal structure (in the absence of the MsbA structure), but the study has been submitted under the 'Tools' heading, so I am not sure this is necessary.

We removed the sentence referencing our unpublished structure of TM287/288, which was solved by the help of a conformation-specific sybody.

The technology works and is easy to use.

We consider this statement provided by our collaborator as a convincing argument for the high efficiency and ease-of-use of the sybody platform.

Reviewer #2:

This manuscript by Zimmermann and colleagues describes a set of three "sybody" libraries based on recently published nanobodies that were successfully used to determine structures of proteins in specific conformations. The authors describe important features and parameters that they optimized to obtain libraries that produce useful binders. The libraries are also validated with multiple targets, ranging from the simple/easy target (MBP) to much more challenging ones, including two eukaryotic SLC transporters. Overall, the libraries performed very well, and the results with the ABC transporter are particularly impressive. The advantages and potential pitfalls, and the reasoning for specific choices are all well described and provide a valuable resource for others considering building or using a similar library.

I could not find the nucleotide mixtures used for the three mixes at the randomized codons anywhere in the manuscript (the legend to Figure 1—figure supplement 2 states "Details are provided in Supplementary results." But no Supplementary results were included, and this was the only reference to such Supplementary results. These mixtures need to be described in the manuscript as they are an important feature of the libraries.

We would like to thank the reviewer for spotting this omission. This information is now placed in the main text (subsection “Establishment of the sybody framework and randomization strategy”). We therefore write in the figure legend of Figure 1—figure supplement 2C: “The rationale behind the three randomization mixtures is provided in the main text”. Figure 1—figure supplement 2C in fact details the exact composition of the three mixtures used for randomization.

The Materials and methods section describing the construction of the vectors and libraries are rather challenging to follow. The authors should consider reorganizing or perhaps reformatting so that the stepwise process is made clearer.

To make the sequences of the vectors fully available, we submitted them to the Addgene plasmid repository. The section on the construction of the libraries may seem a bit overwhelming at first sight, but contains all relevant details required to reproduce it. Furthermore, Table 7 provides an overview on which megaprimers and primers were used to assemble the individual CDRs of the three libraries.

At the end of the Discussion section, the authors state that "The libraries and protocols will be made fully available to academic labs in order to facilitate the spread and further development of the technology." This statement would be much more effective if the relevant scaffold plasmids and other vectors used were available through a service like Addgene and the database numbers were directly included in this manuscript.

We followed this suggestion and made all vectors and sybody scaffold sequences available through Addgene (see also new Table 3).

Reviewer #3:

The work is a logical, incremental improvement in binder generation by integrating several lines of prior studies over the last two decades. Although the developed libraries seem effective and the manuscript provides detailed descriptions of the study, this work does not critically evaluate the technology or provides mechanistic insights into the protein systems studied. The manuscript overstates innovation and impact of this study and makes many unsupported statements.

We agree with the reviewer that library construction and display systems build on previous work. But these are not the main topic of our manuscript. Rather, our study directly addresses a major bottleneck in structural biology and membrane protein biochemistry and its main achievement is the successful selection of binders against purified membrane proteins, which remains a very challenging and often unsuccessful task.

*The authors attribute their successes to three factors, large library size, selection using both ribosome display and phage display, and* in vitro *selection at low temperature and in the presence of inhibitors. However, the authors provide no supporting evidence for these claims. Similarly, the authors imply superiority of their platform over previously published synthetic nanobody libraries with no direct evidence (Discussion section).*

Successful selection outcomes for three different and very difficult membrane protein targets are reported in this publication. To put the statement into perspective, we wish to note that there are no reports of binders in the literature against these targets although the human SLC transporters are key targets of academic research as well as clinical stage drug discovery.

Concerning library size, we consider it as self-evident that larger library sizes result in superior selection outcomes in terms of binder diversity and average binder affinity (under the condition that different numbers of one and the same library are displayed using the same display system, e.g. phage or ribosome display). Therefore, we did not consider it as worthwhile to perform test selection in which we artificially reduced the library size to provide experimental evidence for this claim.

Key to the method presented is that the display system changes from ribosome display in the first round to phage display in consecutive rounds. We convincingly show that single domain antibodies are very efficiently displayed on ribosomes (Figure 1—figure supplement 4). Library sizes were experimentally determined throughout the entire selection process. Due to the use of ribosome display, the maximal number of different sybodies displayed in the first selection round is very high (10^12^). 10^6^ library members are the output of the first selection round, as determined by qPCR. Therefore, a comparatively small phage library (size of around 10^7^ clones) almost completely recovers the diversity of the ribosome display output. The switch to phage display may result in the loss of some binders that are only efficiently displayed on ribosomes, but not on phages. However, in our experience, this does not create a major bias. A large proportion of sybodies selected via three rounds of ribosome display against MBP could be expressed efficiently in *E. coli* for ELISA and protein purification, as we show in Figure 2—figure supplement 1. Switching to phage display has in fact the advantage that we positively select for sybodies that are well expressed in *E. coli*.

Concerning the use of both ribosome and phage display to represent a critical factor, we further support this claim by including additional data in the manuscript in which we describe the gradual improvement of our selection cascade (new Figure 1—figure supplement 6 and description in the main text). The selection cascade evolved as follows:

i) Three rounds of ribosome display against TM287/288 did not result in enrichment nor in any positive hit in ELISA.

ii) Switching to phage display after one round of ribosome display (but not yet altering the chemistry of the immobilization surface) resulted in a few sybody hits against TM287/288, which however could not be purified.

iii) Using in addition solution panning and different surface chemistries resulted in a large number of binders against ABC transporter IrtAB. However, the sybodies were not of high affinity.

iv) The identification and removal of two bottlenecks during the selection process (namely amplification of cDNA after ribosome display and low infection rates of M13 phages) resulted in highly diverse pools containing many high affinity binders against TM287/288 (as shown in Figures 3 and 4).

Concerning selections at 4°C in the presence of inhibitors, we did not state that this was of general importance for our platform, but merely an experimental requirement of the delicate human SLC transporter targets that can be met by a pure *in vitro* selection approach.

Concerning the comparison to other synthetic nanobodies, we did not state superiority of our sybodies over previous approaches. Rather, we make a comparison focusing on the CDR3 lengths and point out that in camelid nanobodies, long CDR3s are tethered via an extended hydrophobic core (as designed in our convex library) and in some cases by the establishment of a second disulfide bond (Discussion section). Biophysical analysis such as thermal unfolding are essentially lacking in previous synthetic nanobody papers. Hence, a direct comparison is not feasible unless we get access to the previously described libraries. However, we are not aware of any publication in the binder field that experimentally compared two libraries from completely independent labs side-by-side. Hence, providing experimental evidence for superiority of one binder library versus another is not state-of-the-art in the binder selection field.

The description of the challenges in binder generation (Introduction) seem inaccurate. Many synthetic antibodies to membrane proteins seem to have been generated by Kossiakoff and colleagues, straight from a synthetic antibody library without "extensive binder screening and purification efforts".

The work generated by the Kossiakoff laboratory is one beautiful example for the generation of conformation-specific binders against some membrane protein targets. However, more than a decade of experience of the three laboratories represented in this publication in generating binders such as SH3 domains or camelid nanobodies in general and DARPins straight from synthetic libraries against membrane proteins (Mittal et al., 2012, Seeger et al., 2012, unpublished negative results from the Geertsma, Dawson, and Seeger labs) shows that some methods are prone to fail against the very difficult and demanding targets such as ABC and SLC transporters. In our hands, such selections always involved extensive binder screening by ELISA and laborious efforts to find monomeric, well-behaved purified binders.

We have toned down the wording in the Introduction by saying, that “extensive binder screening and purification efforts are often required after selection to identify suitable binders, as was for example the case for DARPin selections against the ABC transporters MsbA and LmrCD carried out in our lab (Mittal et al., 2012; Seeger et al., 2012).”

Subsection “The three binding modes of sybodies”. The designs of the three libraries with different paratope topography closely follow the library designs of non-antibody scaffolds (DARPins, Anticalins, Monobodies) over the last decade (PMID 19416843, 22198408, 24513107). This work is particularly similar in concept to PMID 22198408. These precedents should be properly described in this section.

We agree with the reviewer and would like to mention that the initial version of the manuscript already contained citation of PMID 22198408.

To further strengthen this point in we added a sentence to the discussion where we describe paratope topography design in light of previous designs of non-antibody scaffolds (Discussion section).

Establishment of the sybody framework and randomization strategy closely follows the concept developed for synthetic libraries on a single antibody framework where only exposed CDR positions are diversified (PMID 15066433 in 2004 and subsequent papers). Thus, the authors' apparent claim of novelty in the Discussion section is unsupported. Similarly, the use of biased and restricted amino acid diversity with emphasis on Tyr and Ser has been well established (PMID 15306681, 15854651, 16413576, 17420456).

We respectfully disagree with the reviewer. We did not claim in in the Discussion section that the approach is novel. Rather, we explain why we have based our libraries on single antibody frameworks found in published structures to construct libraries of different CDR3 sizes and we explain why it is not ideal to plant CDR3 loops of different sizes onto one and the same scaffold. To improve the clarity for the reader, we added three citations of studies to illustrate that structure-based scaffold design is a successful strategy used by others in the past (Discussion section).

The authors should compare properties of the MBP-binding sybodies with those of many existing binders to MBP (DARPin, monobodies and Fabs). The authors do not mention that high-affinity monobodies have been generated against MBP from extremely simple and small libraries with as few as 10^6^ members (PMID 17420456, 18602117). These monobodies and the reported sybodies appear to share similar binding modes. Conformation-specific antibodies have been generated for MBP directly from a synthetic phage-display library (PMID 21378967). Therefore, the success in generating MBP-binding sybodies does not offer much support for the effectiveness of the library designs or selection methodology. Why do the sybodies have strong preference to the cleft, although the libraries should present distinct topographies. In contrast, the DARPin and Fabs appear to prefer binding outside the cleft (PMID 15097997; PDB 5BJZ, 5BK1, 5BK2).

We agree with the reviewer that the selection outcomes of sybodies against MBP would be an interesting topic to study in much more detail. However, as MBP is a very easy target (due to its high stability and comparably large hydrophilic surface), it merely represents a simple test and benchmarking case towards the goal and focus of our study to pursue membrane protein targets successfully. Because only three structures of homologous sybodies in complex with MBP were solved in our study (see Figure 2—figure supplement 3), we refrained from drawing any general conclusions of preferred sybody epitopes versus epitopes targeted by other binders such as DARPins, Fabs and monobodies. This would require more extensive characterizations, which go beyond the aims and scope of this study.

In the context of our study, the MBP sybodies were important to show that

i) sybodies can be generated against this target with a very simple protocol (i.e. 3 rounds of ribosome display, target immobilization on the same type of beads in every round),

ii) the framework of the convex sybodies containing a tethered CDR3 looks as designed, as evidenced by the MBP-sybody crystal structure,

iii) convex sybodies bind into the cleft of MBP involving all three CDRs and competed with maltose binding.

Because MBP is a very easy target, we believe it is not a surprise that other groups found binders using as few as 10^6^ library members. Therefore, MPB is not suited to improve binder selection strategies and binder libraries aimed at generating binders against delicate membrane proteins. We therefore fully agree with the reviewer’s statement that “the success in generating MBP-binding sybodies does not offer much support for the effectiveness of the library designs or selection methodology.”

It is unclear why the authors did not use nonhydrolyzable ATP analogs in the selection for TM287/288 or for stabilizing its outward-facing state. The authors used a threshold of just threefold in affinity (corresponding to ~0.7 kcal/mol) for designating conformation-specific binders. Binders with such low specificity are not likely to be useful in biophysical and structural applications. Using a more realistic threshold of tenfold, only five clones would be considered specific. The conclusion for this section (subsection “Conformational trapping of a bacterial ABC transporter”) is an overstatement.

As we have demonstrated experimentally in a previous study (Timachi et al., 2017), nonhydrolyzable ATP analogs do not populate the outward-facing state of TM287/288. That’s why we did not use them to stabilize the outward-facing state.

Concerning the definition of state-specific binders, we now applied the 10-fold affinity difference as suggested by the reviewer. Applying this much stricter criterion, we still have 11 state-specific binders as marked in blue in Figure 4 (and not 5 as stated by reviewer #3). The conclusion for this section is therefore not an overstatement.

Much of the Discussion section repeats descriptions of results and thus adds little to the manuscript. They should be omitted or drastically shortened.

The Discussion section puts our sybody selection platform in context with similar *in vitro* selection approaches and the classical nanobody selection approach using immunization with only minor overlap with the results section. We therefore decided to keep this section as it stands.

Concerning the Discussion section: We discuss the different difficulty levels of our selections and put the results in a larger context. We shortened the sections to avoid repetitions from the results section as suggested by reviewer #3.

The authors should include the full sequences of sybodies to GlyT1 and ENT1. For GlyT1, the authors should describe whether clones from a particular library design preferentially bind to one or the other epitope (Figure 6D).

Sequences are provided in Figure 5—figure supplement 1, Figure 6—figure supplement 1 and Figure 6—figure supplement 2.

[Editors' note: the author responses to the re-review follow.]

The manuscript has been significantly improved but there are some remaining issues that need to be addressed before acceptance, as outlined below:

- In the new Figure 1—figure supplement 6, there is a discrepancy, in that the panel C describes analysis of lrtAB, whereas panels A, B, and D describe results for TM287/288, suggesting that the results are not comparable. Ideally TM287/288 would be tested using the same protocol, although, since these data were motivational rather than demonstrative, this is not in our view, essential. Nevertheless, to gloss over the discrepancy raises concerns. The authors could instead mention, e.g., that the results came from a separate study. And/or they could also remove the arrows from the figure, which imply that they came from a single experimental design process.

We agree with the editors that the improvement of our selection protocol ideally would have been conducted using TM287/288 as the only target. However, we wish to point out that TM287/288 and IrtAB are homologues (sequence identity of 27% ) and in our view represent similar difficulty levels for binder selections. Therefore, we do not consider the inclusion of IrtAB as a discrepancy. We have revised the main text (subsection “The sybody selection cascade to tackle membrane protein targets”) and the legend of Figure 1—figure supplement 6 to clarify that IrtAB selections were part of another study and that IrtAB and TM287/288 are homologues, and therefore can substitute for each other for the sake of selection protocol improvements.

We wish to emphasize that the sequence of improvements as shown in Figure 1—figure supplement 6 (involving IrtAB as detour) were not “constructed” for the sake of storytelling, but really occurred in this order in the lab. We therefore consider the improvements shown in Figure 1—figure supplement 6 to be part of a single experimental design process. Therefore, we did not remove the arrows in the figure.

- Please (briefly) expand on the explanation of solution panning and the alternate surface selection strategy within the main flow of the manuscript (subsection “Conformational trapping of a bacterial ABC transporter”).

We briefly explain what is exactly meant with solution panning and the alternate surface selection strategy in the main text (now subsection “The sybody selection cascade to tackle membrane protein targets”).
